# TNF-α contributes to sarcopenia through caspase-8/caspase-3/GSDME-mediated pyroptosis

**DOI:** 10.1038/s41420-023-01365-6

**Published:** 2023-02-24

**Authors:** Jingying Wu, Siming Lin, Weixiao Chen, Guili Lian, Weibin Wu, Ai Chen, Mohammad Ismail Hajary Sagor, Li Luo, Huajun Wang, Liangdi Xie

**Affiliations:** 1 Department of Geriatrics, The First Affiliated Hospital of Fujian Medical University, 20 Chazhong Road, Fuzhou, Fujian 350005 People’s Republic of China; 2 Fujian Hypertension Research Institute, The First Affiliated Hospital of Fujian Medical University, Fuzhou, People’s Republic of China; 3 Clinical Research Center for Geriatric Hypertension Disease of Fujian province, The First Affiliated Hospital of Fujian Medical University, Fuzhou, People’s Republic of China; 4 Branch of National Clinical Research Center for Aging and Medicine, The First Affiliated Hospital of Fujian Medical University, Fuzhou, Fujian Province People’s Republic of China

**Keywords:** Cell death, Ageing

## Abstract

Sarcopenia has become a leading cause of disability and mortality in the elderly. It has been reported that programmed cell death (PCD) is associated with the development of sarcopenia that is characterized by reduction of muscle fiber size and number. TNF-α is also validated to play a prominent role in sarcopenia through its complex signaling pathways including cell death signaling. However, it is still unclear whether TNF-α contributes to sarcopenia by mediating pyroptosis, one type of PCD. Here, we first established naturally aged mice with sarcopenia model and confirmed an inflammatory state represented by TNF-α in aged mice. Evidence of GSDME-mediated pyroptosis and activation of apoptotic caspase-8/-3 were also found in skeletal muscle cells of aged mice with sarcopenia. We demonstrated that TNF-α triggered GSDME-mediated pyroptosis in myotubes through activating caspase-8 and caspase-3 by using caspase-8 and caspase-3 inhibitors. Comparing the activation of caspase-8 and GSDME expression between TNF Complex IIa and TNF Complex IIb, TNF-α was found to be more inclined to assemble TNF Complex IIb in activating caspase-8 and triggering pyroptosis. Moreover, pyroptotic myotubes were validated to result in decreased expression of MHC1 and finally loss of myotubes by knockdown of GSDME. Our work reveals a novel mechanism that TNF-ɑ/caspase-8/caspase-3/GSDME signaling-mediated pyroptosis contributes to the development of sarcopenia. Caspase-3/GSDME signaling-mediated pyroptosis may be a promising therapeutic target for sarcopenia.

## Introduction

Sarcopenia is a multifactorial disease characterized by age-related decline in skeletal muscle mass, strength, and physical performance [1]. Histologically, the major pathological characteristic of sarcopenia is a decline in muscle cell size and number that results in the decrease in muscle mass and strength [2]. However, the molecular mechanisms of sarcopenia remain incompletely understood.

Programmed cell death (PCD) refers to a type of strictly regulated cell death, including apoptosis, pyroptosis, necroptosis, ferroptosis, and autophagy [3]. It has been reported that PCD is responsible for cell loss in several tissues during aging, including skeletal muscle [4]. Hence, the loss of skeletal muscle fibers during aging, as critical characteristic of sarcopenia, is possibly attributed to PCD [4–6]. Both apoptosis and autophagy have been revealed to involve in the development of sarcopenia. [7–9]. Recently, ferroptosis was also found in skeletal muscle cells of aged mice [10]. However, it remains unclear whether other types of PCD such as pyroptosis also occurs in skeletal muscle cells and contributes to sarcopenia.

Morphologically, pyroptosis manifests as the formation of pores in the cell membrane, followed by cell swelling with membrane rupture and finally cell death [11]. The executor of pyroptosis is a family of Gasdermins (GSDMs), including GSDMA, GSDMB, GSDMC, GSDMD, GSDME, and PJVK [12]. All GSDMs except PJVK is cleaved by activated caspase to release the N-terminal domain. The intracytoplasmic N-terminal domain translocates to the membrane and oligomerizes to form pores in the cell membrane, allowing inflammatory contents to release and ultimately executing cell death [13]. Initially, it is reported that pyroptosis is triggered through two pathways that eventually cleave GSDMD [14]: the classical caspase-1-dependent pathway, and the nonclassical caspase-4/-5/-11-dependent pathway. Recent studies have also demonstrated that GSDME is cleaved by activated caspase-3 to execute pyroptosis [15].

Interestingly, interconnectivity exists between different types of PCD [3, 16]. A typical example is that activated caspase-3 cleaves GSDME to induce pyroptosis, initiating the switching of apoptosis to pyroptosis [17]. Tumor necrosis factor alpha (TNF-α), as an inflammatory factor, is considered to play a prominent role in the interconnection of different types of PCD due to its complex cell death signaling [18]. TNF-α is released by inflammatory cells, as well as skeletal muscle cells [19]. TNF-α exerts complex cell death signaling by binding to TNF receptor 1 (ref. [20]). The type of cell death is dependent on the assembly of TNF complexes, including TNF Complex IIa, IIb, and IIc. Both TNF Complex IIa and IIb activate caspase-8 and downstream caspase-3 to trigger apoptosis, further inducing GSDME-mediated pyroptosis. Additionally, TNF Complex IIc triggers necroptosis by a receptor-interacting protein kinase 3 (RIPK3)-dependent mechanism [21].

Chronic low-grade inflammation was confirmed to develop with age in the elderly, with inflammatory cells infiltrating among the skeletal muscle cells and releasing pro-inflammatory cytokines such as TNF-α to cause muscle damage [22]. TNF-α has been validated to participate in the pathogenesis of sarcopenia through its complex intracellular signals [6, 23, 24]. However, it is unclear whether TNF-α contributes to sarcopenia by inducing myofibers death through the interconnection of different types of PCD, such as the crosstalk between apoptosis and pyroptosis.

In this paper, we established naturally aged mice with sarcopenia model to observe the inflammatory state represented by TNF-α and to identify whether pyroptosis occurs in the skeletal muscle cells. TNF-α-stimulated myotube is a well-known in vitro study on sarcopenia [25]. We thus used TNF-α as a stimulator of myotubes to further investigate mechanism underlying how TNF-α induces sarcopenia by regulating pyroptosis and the possible crosstalk between apoptosis and pyroptosis.

## Results

### Murine model of sarcopenia presented an inflammatory state with an elevated level of TNF-α that was associated with loss of skeletal muscle mass and grip strength

**Fig. 1:**
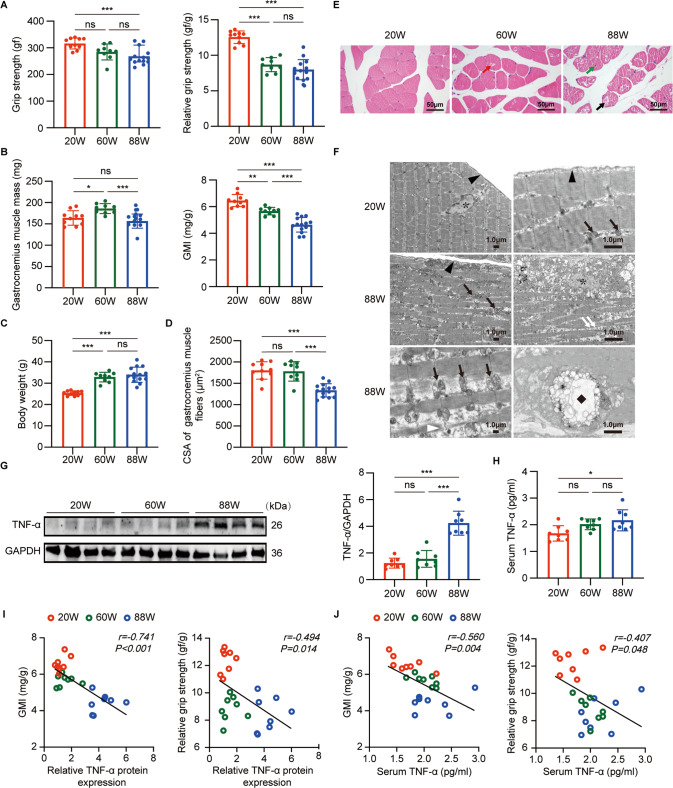
Murine model of sarcopenia presented with high level of serum TNF-α and TNF-α overexpression in gastrocnemius muscle (GM) that were associated with loss of skeletal muscle mass and grip strength. **A**–**D** Grip strength, relative grip strength, GM mass, gastrocnemius muscle index (GMI), body weight and cross-sectional area (CSA) of GM fibers in mice at different ages. **E** HE staining of GM in mice at different ages. Red arrow indicates cellular vacuolation of GM fibers in mice aged 60 weeks, black arrow indicates cellular vacuolation of GM fibers in mice aged 88 weeks, and green arrow indicates centralized nucleus of GM cells in mice aged 88 weeks. Scale bar: 50 μm. **F** transmission electron microscope (TEM) micrographs of GM in mice aged 20 weeks and 88 weeks. Black arrows indicate mitochondria, white arrows indicate lipid droplets, black triangles indicate cell membrane, white triangle indicates myofilament lysis, asterisks indicate nucleus, diamond indicates multivesicular bodies. Scale bar: 1.0 μm. **G** immunoblot of TNF-α expression in GM of mice at different ages. The relative expression level of TNF-α normalized to GAPDH based on densitometric analysis of immunoblot. **H** ELISA assay of serum TNF-α in mice at different ages. **I** correlation analysis of the relative expression level of TNF-α with relative grip strength and GMI in mice. **J** correlation analysis of serum TNF-α level with relative grip strength and GMI in mice. Data are expressed as mean ± SD. *P < 0.05, **P < 0.01, ***P < 0.001, ns: no significance. **A**–**F** Mice aged 20 weeks: *n* = 10 mice, mice aged 60 weeks: *n* = 9 mice, mice aged 88 weeks: *n* = 14 mice. **G**–**J**, *n* = 8 mice per group.

The gastrocnemius muscle index (GMI), absolute, and relative grip strength of aged mice were significantly lower than those of young mice, although there was no significant difference in the absolute mass of gastrocnemius muscle (GM) between the two groups. Meanwhile, the absolute mass of GM and GMI of aged mice were dramatically lower than those of middle-aged mice, whereas no significant differences in the absolute and relative grip strength were found between the two groups (Fig. 1A, B). The cross-sectional area (CSA) of muscle fibers was significantly decreased in aged mice compared with that in young and middle-aged mice (Fig. 1D). Hematoxylin and eosin (HE) staining showed that the muscle fibers of aged mice exhibited shrunken shape, cellular vacuolation (Fig. 1E black arrow), and centralized nucleus (Fig. 1E green arrow). Scattered cellular vacuolation was also found in the muscle fibers of middle-aged mice (Fig. 1E red arrow). Transmission electron microscope (TEM) showed that the muscle cells of aged mice exhibited disordered sarcomere, myolysis (Fig. 1F, white triangle), nuclear degeneration (Fig. 1F, asterisk), mitochondrial enlargement (Fig. 1F, black arrow), as well as lipid infiltration (Fig. 1F, white arrow) and multivesicular bodies (Fig. 1F, black diamond). The above results indicated a successful establishment of murine model of sarcopenia.

The expression of TNF-α was considerably higher in GM of aged mice than that in young and middle-aged mice (Fig. 1G). Consistently, the level of serum TNF-α in the aged mice was significantly higher than that in young mice (Fig. 1H). Furthermore, Pearson’s correlation coefficient showed that both level of serum TNF-α and expression of local TNF-α correlated negatively with GMI and relative grip strength (Fig. 1I, J). These results strongly supported a possible causative role of TNF-α in age-related decline in skeletal muscle mass and strength.

### The skeletal muscle of aged mice with sarcopenia underwent GSDME-mediated pyroptosis accompanied by activation of apoptosic caspase-8/-3

**Fig. 2:**
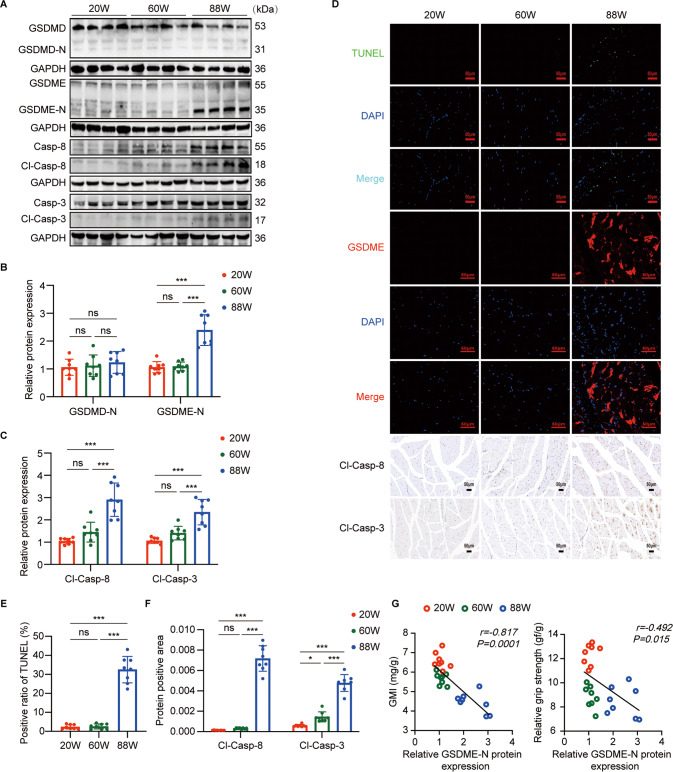
GSDME-mediated pyroptosis and activation of apoptosic caspase-8/-3 were present in GM of aged mice with sarcopenia. **A** Representative immunoblots of GSDMD, N terminus of GSDMD(GSDMD-N), GSDME, N terminus of GSDME(GSDME-N), caspase (Casp)-8, cleaved-Casp(Cl-Casp)-8, Casp-3 and Cl-Casp-3 in GM of mice at different ages. **B**, **C** Relative expression levels of GSDMD-N, GSDME-N, Cl-Casp-8, and Cl-Casp-3 normalized to GAPDH based on densitometric analysis of immunoblots. **D** TUNEL staining, immunofluorescence staining of GSDME and immunohistochemical staining of Cl-Casp-8 and Cl-Casp-3 in GM of mice at different ages. Scale bar: 50 μm. **E** positive rates of TUNEL staining in the mice at different ages. **F** quantitation of immunohistochemical analyses for Cl-Casp-8 and Cl-Casp-3. **G**, correlation analysis of the relative expression level of GSDME-N with relative grip strength and GMI in mice. Data are expressed as mean ± SD. *P < 0.05, ***P < 0.001, ns: no significance. *n* = 8 mice per group.

Since the common protein executors for pyroptosis are GSDMD and GSDME, we detected the expression of GSDMD and GSDME in GM of mice at different ages. Western blot showed that GSDMD was not cleaved in all of three groups, while GSDME was significantly cleaved in the aged mice compared with that in young and middle-aged mice (Fig. 2A, B). Meanwhile, immunofluorescence of GSDME in GM of aged mice also revealed a dramatically higher expression (Fig. 2D). These results suggested that GSDME-mediated pyroptosis occurs in the skeletal muscle of aged mice with sarcopenia. It was further supported that the percentage of positive TUNEL staining in aged mice was higher than in both young and middle-aged mice (Fig. 2D, E). Moreover, the correlation analysis showed that the N terminus of GSDME (GSDME-N) expression correlated negatively both with relative grip strength and GMI (Fig. 2G). Our data suggested that GSDME-mediated pyroptosis involves in an age-related decline in skeletal muscle mass and strength. In addition, the western blot revealed higher expression of both cleaved-caspase-8 and cleaved-caspase-3 in GM of aged mice compared with those in GM of young and middle-aged mice (Fig. 2A, C). Consistently, immunohistochemical staining also showed significantly increased expression of cleaved-caspase-8 and cleaved-caspase-3 in GM of aged mice (Fig. 2D, F). These results indicated that the apoptotic caspase-8/-3 is activated in GM of aged mice with sarcopenia.

### TNF-α induced GSDME-mediated pyroptosis in C2C12 myotubes

**Fig. 3:**
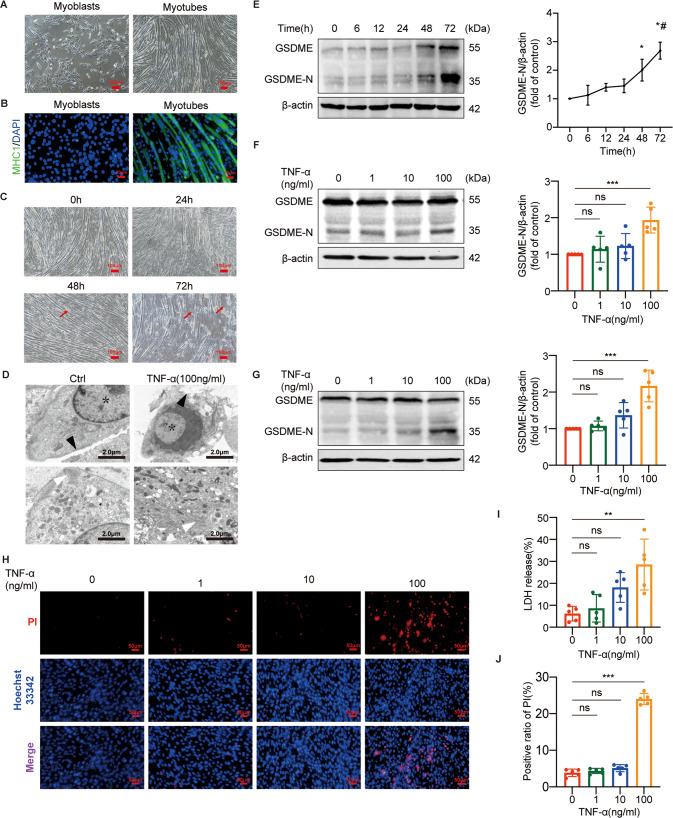
TNF-α induced GSDME-mediated pyroptosis in C2C12 myotubes. **A** Morphology of C2C12 myoblasts and myotubes. Scale bar:100 μm. **B** immunofluorescence staining of myoblasts and myotubes for myosin heavy chain 1 (MHC1, green). Nuclei are stained with DAPI. Scale bar: 50 μm. **C** representative microscopic images were taken after myotubes were treated with TNF-α (100 ng/ml) at the indicated time points. Red arrows indicate cell death. Scale bar: 100 μm. **D** TEM micrographs were taken after myotubes were treated with TNF-α (100 ng/ml) for 72 h. Scale bar: 2.0μm. black triangles indicate cell membrane, white triangles indicate myofilaments, and asterisks indicate nucleus. **E** immunoblots of GSDME and GSDME-N in myotubes treated with TNF-α (100 ng/ml) at the indicated time points. The relative expression level of GSDME-N normalized to β-actin based on densitometric analysis of immunoblot. *P < 0.05 vs. 0 h, ^#^*P* < 0.05 vs. 48 h. **F**, **G** immunoblots of GSDME and GSDME-N in myotubes treated with different concentrations (0, 1, 10, or 100 ng/ml) of TNF-α for 48 h (**F**) or 72 h (**G**). Relative expression levels of GSDME-N normalized to β-actin based on densitometric analysis of immunoblots. **H**, **I** cell death was determined by staining with Hoechst 33342/ propidium iodide (PI) (**H**) and measuring lactate dehydrogenase (LDH) release into the cell culture supernatant (**I**) after myotubes were treated with different concentrations (0, 1, 10 or 100 ng/ml) of TNF-α for 72 h. Scale bar: 50 μm. **J** Quantitation of the positive ratio of PI. Data are expressed as mean ± SD. **P < 0.01, ***P < 0.001, ns: no significance. *n* = 5 independent experiments.

C2C12 myoblasts are mononuclear with spindle shape, whereas C2C12 myotubes are multi-nuclear with elongated morphology (Fig. 3A). The differentiated myotubes were morphologically identified by immunofluorescence with a monoclonal antibody against MHC1, a specific biomarker of myotube (Fig. 3B). To determine optimal timing, we incubated myotubes with TNF-α (100 ng/ml) at different time points (0, 6, 12, 24, 48, 72 h). The cleavage of GSDME was induced by TNF-α after 48 h treatment, reaching the maximum at 72 h (Fig. 3E). Meanwhile, the myotubes were incubated with different concentrations of TNF-α (0,1,10,100 ng/ml) for 48 h and 72 h, respectively. The peak effect of TNF-α on cleaving GSDME was shown at a concentration of 100 ng/ml (Fig. 3F, G). Consistently, the swelling and death of myotubes were observed after 48 h TNF-α treatment and more pronounced after 72 h. (Fig. 3C). TEM also revealed membrane disruption and nuclear degeneration in TNF-α-treated myotubes (Fig. 3D). In addition, both the positive ratio of propidium iodide (PI) and rate of lactate dehydrogenase (LDH) release increased with the concentration of TNF-α, indicating cell death with extensive lysis and a difference from apoptosis-induced cell death. Overall, our data demonstrated that TNF-α induces GSDME-dependent pyroptosis in myotubes.

### TNF-α induced GSDME-mediated pyroptosis through the activation of caspase-8 and caspase-3 in C2C12 myotubes

**Fig. 4:**
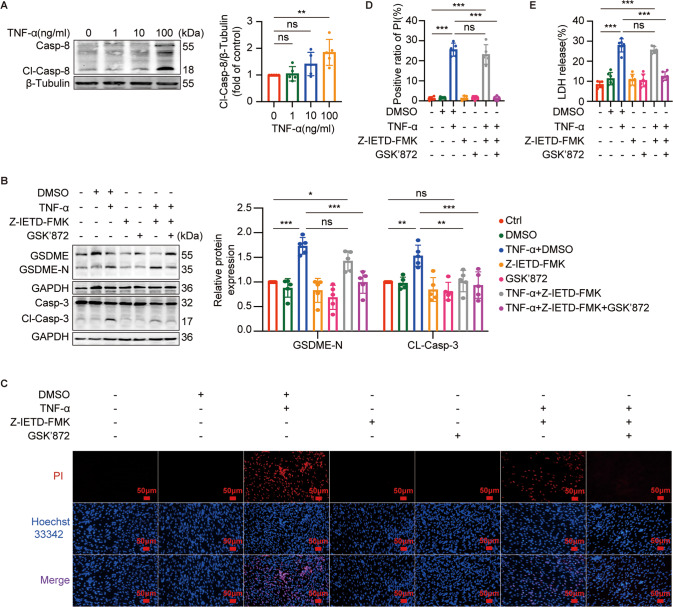
Activation of Casp-8 was required for TNF-α-induced pyroptosis in C2C12 myotubes. **A** immunoblots of Casp-8 and Cl-Casp-8 in myotubes treated with different concentrations (0, 1, 10, or 100 ng/ml) of TNF-α for 72 h. Relative expression levels of Cl-Casp-8 normalized to β-Tubulin based on densitometric analysis of immunoblots. **B**–**E** Myotubes were pretreated with GSK'872 (1 μM) for 30 min and then Z-IETD-FMK (50 μM) for 1 h followed by treatment with TNF-α (100 ng/ml) for 72 h. The expression of GSDME, GSDME-N, Casp-3, and Cl-Casp-3 in myotubes were immunoblotted and analyzed based on densitometric analysis of immunoblots (**B**). Cell death was determined by staining with Hoechst 33342/PI (**C**) and measuring LDH release into the cell culture supernatant (**E**) after myotubes treatments. Scale bar: 50 μm. **D** Quantitation of positive ratio of PI. Data are expressed as mean ± SD. *P < 0.05, **P < 0.01, ***P < 0.001, ns: no significance. *n* = 5 independent experiments.

**Fig. 5:**
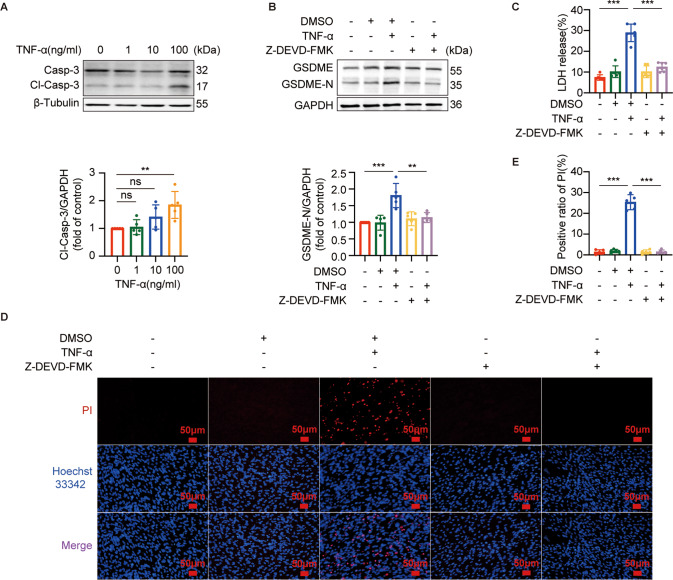
Activation of Casp-3 was required for TNF-α-induced pyroptosis in C2C12 myotubes. **A** immunoblots of Casp-3 and Cl-Casp-3 in myotubes treated with different concentrations (0, 1, 10, or 100 ng/ml) of TNF-α for 72 h. Relative expression levels of Cl-Casp-3 normalized to β-Tubulin based on densitometric analysis of immunoblots. **B**–**E** myotubes were pretreated with Z-DEVD-FMK (50 μM) for 1 h followed by treatment with TNF-α (100 ng/ml) for 72 h. The expressions of GSDME and GSDME-N in myotubes were immunoblotted and analyzed based on densitometric analysis of immunoblots (**B**). Cell death was determined by measuring LDH release into the cell culture supernatant (**C**) and staining with Hoechst 33342/PI (**D**) after myotubes treatments. Scale bar: 50 μm. **E** Quantitation of positive ratio of PI. Data are expressed as mean ± SD. **P < 0.01, ***P < 0.001, ns: no significance. *n* = 5 independent experiments.

Next, we investigated whether TNF-α induced GSDME-mediated pyroptosis in myotubes through caspase-3 and upstream caspase-8. Both caspase-8 and caspase-3 were activated with the concentration of TNF-α ranging from 0 to 100 ng/ml in myotubes (Figs. 4A and 5A). We used Z-IETD-FMK, a specific inhibitor of caspase-8, to confirm the role of caspase-8 in TNF-α-induced pyroptosis. Since activated caspase-8 in TNF-α-induced cell death signaling can block necroptosis by inhibiting RIPK1 and RIPK3, crucial factors of necroptosis, finally leading to the occurrence of apoptosis [26]. Therefore, necroptosis may be elicited if the activation of caspase-8 is inhibited by Z-IETD-FMK. To exclude the interference of necroptosis in the following cell death experiments, we also used GSK’872, an inhibitor of RIPK3, to block necroptosis in the subsequent experiments. As shown in Fig. 4, the cleavage of GSDME(Fig. 4B), positive PI staining (Fig. 4C, D), and increased LDH release (Fig. 4E) induced by TNF-α were all inhibited by the addition of Z-IETD-FMK and GSK’872, suggesting that activation of caspase-8 was required for TNF-α-induced pyroptosis. Noteworthy, in the presence of Z-IETD-FMK alone, the effects of TNF-α on myotubes were still prominent. Additionally, the activation of caspase-3 induced by TNF-α was inhibited by Z-IETD-FMK (Fig. 4B), conforming that caspase-8 is in upstream of caspase-3. We also used Z-DEVD-FMK, a specific inhibitor of caspase-3, to validate the involvement of caspase-3 in GSDME-mediated pyroptosis induced by TNF-α. the cleavage of GSDME (Fig. 5B), increased LDH release (Fig. 5C), and positive PI staining (Fig. 5D, E) induced by TNF-α were all reversed by the addition of Z-DEVD-FMK.

### TNF Complex IIb but not Complex IIa enables activation of caspase-8 and subsequent occurrence of caspase-3/GSDME-mediated pyroptosis in TNF-α-treated myotubes

**Fig. 6:**
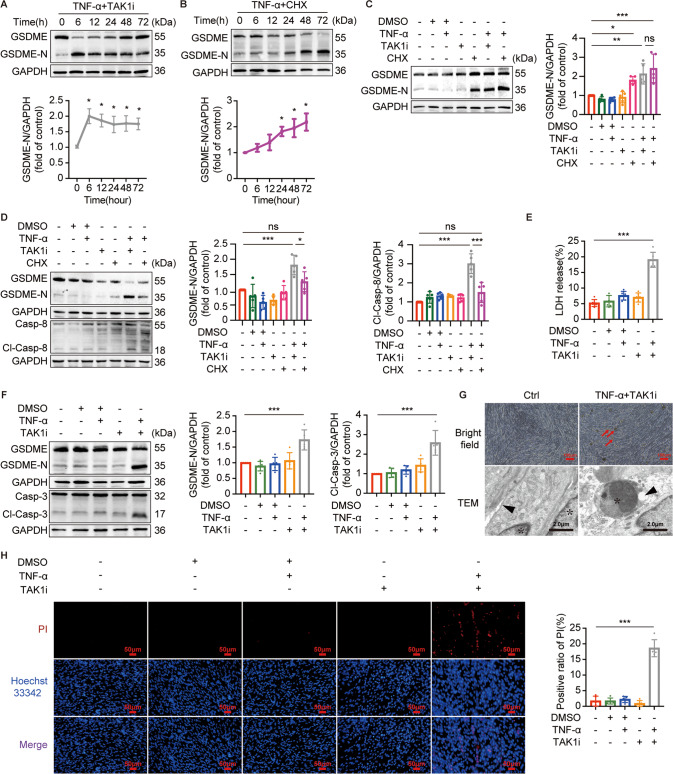
TNF Complex IIb but not Complex IIa enables TNF-α-induced caspase-8 activation and subsequent caspase-3/GSDME-mediated pyroptosis occurrence. **A**, **B** immunoblots of GSDME and GSDME-N in myotubes treated with TNF-α (100 ng/ml) + TAK1 inhibitor (TAK1i, 1 μM) (**A**) or TNF-α (100 ng/ml) +cycloheximide (CHX, 10 μg/ml) (**B**) at the indicated time points. Relative expression level of GSDME-N normalized to GAPDH based on densitometric analysis of immunoblot. *P < 0.05 vs. 0 h. **C**, **D** immunoblots of GSDME, GSDME-N, Casp-8, and Cl-Casp-8 in myotubes treated with TNF-α (100 ng/ml) + TAK1i (1 μM) or TNF-α (100 ng/ml) + CHX (10 μg/ml) for 6 h (**D**) or 24 h (**C**). Relative expression level of GSDME-N and Cl-Casp-8 normalized to GAPDH based on densitometric analysis of immunoblot. **E**–**H** myotubes were treated with TNF-α (100 ng/ml) +TAK1i (1 μM) for 6 h. Cell death was determined by measuring LDH release into the cell culture supernatant (**E**) and staining with Hoechst 33342/PI (**H**) after myotubes treatments. Scale bar: 50 μm.The expressions of GSDME, GSDME-N, Casp-3, and Cl-Casp-3 in myotubes were immunoblotted and analyzed based on densitometric analysis of immunoblots (**F**). Representative optical microscope images and TEM micrographs were taken after myotubes were treated with or without TNF-α + TAK1i (**G**). Red arrows indicate cell death, black triangles indicate cell membrane, white triangles indicate myofilaments, and asterisks indicate nucleus. Scale bar: 100μm for optical microscope images. Scale bar: 2.0 μm for TEM micrographs. Data are expressed as mean ± SD. *P < 0.05, **P < 0.01, ***P < 0.001, ns: no significance. *n* = 5 independent experiments.

TNF-α can trigger the activation of caspase-8 either by the assembly of TNF Complex IIa or by TNF Complex IIb [27]. However, it is unclear which one of TNF Complex activates caspase-8 in response to TNF-α-induced pyroptosis in myotubes. Usually, TNF Complex IIa is assembled when cells are simultaneously treated with TNF-α and protein synthesis inhibitor cycloheximide (CHX). Alternatively, TNF Complex IIb is assembled when cells are simultaneously treated with TNF-α and TGF-β-activated kinase 1 inhibitor (TAK1i) 5Z-7-Oxozeaenol [28]. Hence, as in a previous study [27], we compared the activation of caspase-8 and GSDME-N expression in myotubes stimulated with TNF-α + CHX or TNF-α + TAK1i to induce assembly of TNF Complex IIa or TNF Complex IIb respectively. As shown in Fig. 6, TNF-α + TAK1i increased GSDME-N expression with a time course, reaching the maximum effect as early as 6 h after stimulation (Fig. 6A). GSDME-N expression in myotubes stimulated by TNF-α + CHX also increased with a time course but reached a significant effect after 24 h of stimulation (Fig. 6B). Furthermore, the expression of GSDME-N was dramatically higher in TNF-α + TAK1i-treated myotubes compared to TNF-α + CHX-treated myotubes at 6 h, but no significant difference was found at 24 h of stimulation (Fig. 6C, D). Consistently, caspase-8 was considerably activated by TNF-α + TAK1i stimulation as compared with TNF-α + CHX stimulation at 6 h (Fig. 6D). These data indicated that TNF-α may be more inclined to induce the activation of caspase-8 and subsequent GSDME-mediated pyroptosis through the assembly of TNF Complex IIb in myotubes. Additionally, the activation of caspase-3 in myotubes was also triggered after the stimulation of TNF-α + TAK1i (Fig. 6F). Moreover, cell death induced by TNF-α + TAK1i was validated by the morphological characteristic of cell swelling, membrane disruption and nuclear degeneration (Fig. 6G), as well as the significantly increased rate of LDH release (Fig. 6E) and positive ratio of PI (Fig. 6H). Collectively, our data demonstrated that TNF-α induces the activation of caspase-8/-3 and subsequent GSDME-mediated pyroptosis through assembly of TNF Complex IIb in myotubes.

### TNF-α promotes sarcopenia through TNF complex IIb/caspase-8/caspase-3/GSDME-mediated pyroptosis in myotubes

C2C12 cell has been recognized as a well-documented in vitro cell line for mimicking molecular mechanisms of sarcopenia [25, 29]. Differentiated C2C12 myotubes possess features that are similar to human myotubes [29] and carry the same genes involved in muscle development and contraction: MYH1 that encodes MHC1, main composition of type IIX fast fibers, and MYH4 that encodes MHC4, main composition of type IIB fast fibers [30]. Aging mainly affects type II fast fibers, especially type IIA and IIX fibers [31]. Based on previous in vitro studies on sarcopenia [32], we then used MHC1 as a marker of the in vitro study of sarcopenia to explored whether TNF-α-induced sarcopenia is attributed to GSDME-mediated pyroptosis.

**Fig. 7:**
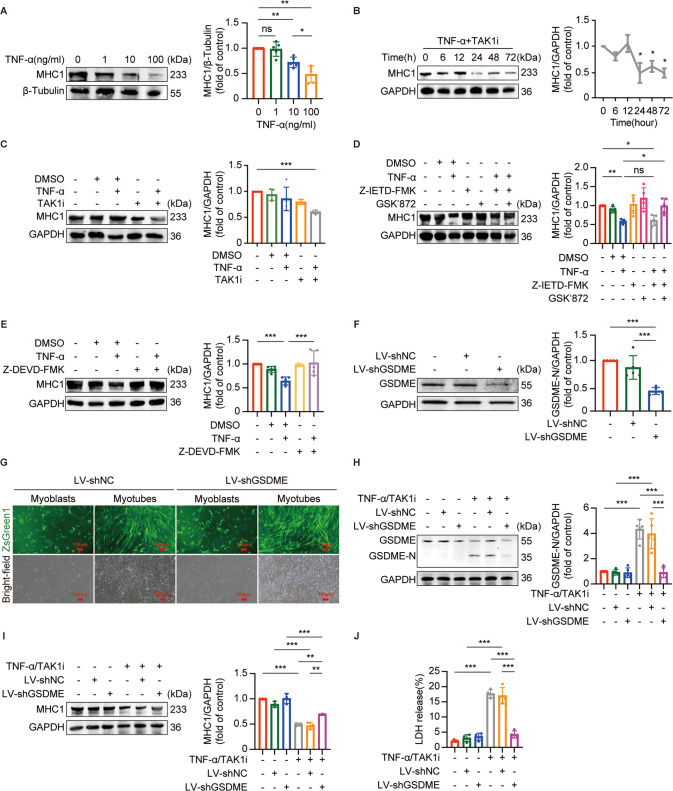
TNF Complex IIb/caspase-8/caspase-3/GSDME-mediated pyroptosis is involved in TNF-α-induced sarcopenia in myotubes. **A** immunoblot of myosin heavy chain 1 (MHC1) in myotubes treated with different concentrations (0, 1, 10, or 100 ng/ml) of TNF-α for 72 h. Relative expression levels of MHC1 normalized to β-Tubulin based on densitometric analysis of immunoblots. **B** immunoblots of MHC1 in myotubes treated with TNF-α (100 ng/ml) +TAK1i (1 μM) at the indicated time points. Relative expression level of MHC1 normalized to GAPDH based on densitometric analysis of immunoblot. *P < 0.05 vs. 0 h. **C** immunoblots of MHC1 in myotubes treated with TNF-α (100 ng/ml) + TAK1i (1 μM) for 24 h. Relative expression level of MHC1 normalized to GAPDH based on densitometric analysis of immunoblot. **D** myotubes were pretreated with GSK'872 (1 μM) for 30 min and then Z-IETD-FMK (50 μM) for 1 h followed by treatment with TNF-α (100 ng/ml) for 72 h. The expression of MHC1 in myotubes were immunoblotted and analyzed based on densitometric analysis of immunoblots. **E** myotubes were pretreated with Z-DEVD-FMK (50 μM) for 1 h followed by treatment with TNF-α (100 ng/ml) for 72 h. The expressions of MHC1 in myotubes were immunoblotted and analyzed based on densitometric analysis of immunoblots. **F**–**J** myoblasts were transfected with GSDME knockdown lentivirus (LV-shGSDME) and then induced to differentiate into myotubes. Knockdown efficiency was examined by immunoblotting of GSDME (**F**). Morphologies of myoblasts and myotubes were observed by fluorescence microscopy (**G**). Scale bar: 100 μm. After treating myotubes with TNF-α (100 ng/ml) +TAK1i (1 μM) for 6 h, immunoblots of GSDME and GSDME-N (**H**) and detection of LDH release (**J**) in myotubes were performed. The expressions of MHC1 in myotubes treated with TNF-α (100 ng/ml) + TAK1i (1 μM) for 24 h were immunoblotted and analyzed based on densitometric analysis of immunoblots (**I**). Data are expressed as mean ± SD. *P < 0.05, **P < 0.01, ***P < 0.001, ns: no significance. **A**–**H** *n* = 5 independent experiments. **I**, **J** *n* = 4 independent experiments.

The expression of MHC1 declined with the concentration of TNF-α in myotubes (Fig. 7A), indicating that TNF-α can successfully induce sarcopenia in vitro. The expression of MHC1 reduced with a time course after TNF-α + TAK1i stimulation, reaching the minimal expression as early as 24 h (Fig. 7B, C), suggesting that TNF-α induced sarcopenia through TNF Complex IIb in myotubes. Furthermore, the reduced level of MHC1 in TNF-α-treated myotubes was reversed by the addition of Z-IETD-FMK and GSK’872. Consistent with the effect on cleavage of GSDME, Z-IETD-FMK alone was not enough to reserve the effect of TNF-α on the expression of MHC1 (Fig. 7D). Additionally, the presence of Z-DEVD-FMK also reversed the level of MHC1 reduced by TNF-α (Fig. 7E). These data validated that TNF-α induces sarcopenia via TNF Complex IIb/caspase-8/caspase-3.

To further clarify the contribution of GSDME-mediated pyroptosis to TNF-α-induced sarcopenia, we transfected myoblasts either with lentivirus encoding shRNA targeting GSDME(LV-shGSDME) or empty lentiviral vector (LV-shNC) according to the optimal MOI for infection (Supplemental Fig. A, B) and validated the knockdown efficiency by immunoblotting of GSDME (Fig. 7F). Thereafter, transfected myoblasts were successfully differentiated into myotubes to conduct the following experiments (Fig. 7G and Supplemental Fig. C). As shown in Fig. 7H, J, knockdown of GSDME inhibited the cleavage of GSDME and LDH release in TNF-α + TAK1i-stimulated myotubes, suggesting inhibition of GSDME-mediated pyroptosis by silencing GSDME. Furthermore, knockdown of GSDME also reserved the decreased expression of MHC1 in myotubes induced by TNF-α + TAK1i (Fig. 7I). However, it was worth noting that knockdown of GSDME, to some extent, partially increase the expression of MHC1 as compared with untreated myotubes (Fig. 7I). Taken together, our data indicated that GSDME-mediated pyroptosis contributes to TNF-α-induced sarcopenia.

## Discussion

In the current study, we identified that aged mice with sarcopenia presented a chronic low-grade inflammation with a high level of serum TNF-α and local TNF-α overexpression in GM, accompanied by evidence of GSDME-mediated pyroptosis and activation of apoptotic caspase-8/-3 in skeletal muscle cells. We demonstrated that TNF-α triggered GSDME-mediated pyroptosis in myotubes by activating caspase-8 and caspase-3 through TNF Complex IIb. Furthermore, pyroptotic myotubes resulted in decreased expression of MHC1 and loss of myotubes, finally leading to sarcopenia. Our study highlights the pathophysiological role of pyroptosis mediated by TNF-ɑ/caspase-8/caspae-3/GSDME signaling in the development of sarcopenia.

According to the latest diagnostic criteria of the European Working Group on Sarcopenia in Older People in 2019 [33], low muscle mass and strength are the basis for diagnosing sarcopenia. However, up to date, there are no diagnostic criteria for sarcopenic animals. Consequently, we evaluated the establishment of a natural aging mouse model of sarcopenia based on muscle mass, grip strength, and histomorphological characteristics of skeletal muscle. Consistent with previous studies on sarcopenia [34, 35], we observed a dramatic decline in grip strength, relative grip strength, GMI, and CSA of skeletal muscle fibers in aged mice, as well as degeneration in skeletal muscle cells by HE staining and TEM, indicating a successful construction of the sarcopenic animal model. Notably, the skeletal muscle changes in middle-aged mice were different from the results of another study that showed early aging manifestations in skeletal muscle of 12-month-old mice such as intranuclear migration [36]. Nevertheless, the middle-aged mice in our study exhibited lower relative muscle strength and GMI than young mice, as well as cellular vacuolation in muscle fibers, also suggesting signs of aging in skeletal muscles.

We further found a high level of serum TNF-α and local TNF-α overexpression in GM of aged mice, supporting the commonly recognized view that chronic low-grade inflammation is a hallmark feature of aging [37], as well as sarcopenia [38]. An increased serum level of TNF-α has been regarded as an important inflammatory marker associated with sarcopenia [39]. It is known that the local TNF-α in skeletal muscles is secreted not only by the increased infiltration of macrophage into muscles [38] but also by the aging muscle cells [40]. Both serum and local TNF-α were inversely correlated with GMI and relative muscle strength in our study, suggesting a role of TNF-α in the pathogenesis of sarcopenia. Therefore, we used TNF-α to stimulate myotubes in vitro for exploring underling mechanisms, which has been recognized as a classical in vitro study on sarcopenia [25].

Muscle cell death may be the key factor for the loss of skeletal muscle fibers during aging [6]. We observed that only GSDME but not GSDMD was cleaved in the GM of aged mice, along with high positive ratio of TUNEL, suggesting that skeletal muscle cells of aged mice underwent GSDME-mediated pyroptosis. Recent studies demonstrated that activated caspase-3 is the initiator of pyroptosis, and GSDME is the “switch” between apoptosis and pyroptosis [17]. GSDME-high-expressed cells undergo pyroptosis following the activation of caspase-3, while GSDME-low-expressed cells undergo apoptosis [41]. Therefore, the expression of GSDME determines whether cells undergo apoptosis or pyroptosis. GSDME is expressed in various tissues including skeletal muscle [42]. In our study, the expression of GSDME-N in myotubes increased with the TNF-α treatment in a dose- and time-dependent manner, as did the LDH release rate and PI positive rate. Meanwhile, morphological features of pyroptosis were also observed in TNF-α-treated myotube. Our data strongly indicated that TNF-α triggers GSDME-mediated pyroptosis in myotubes, due to the high expression of GSDME in myotubes.

We observed activation of both caspase-8 and downstream caspase-3 in the GM of aged mice, consistent with the activation of caspase-8 and caspase-3 in TNF-α-treated myotubes, indicating that TNF-α could also activate the apoptosic caspase-8 and caspase-3 in aging skeletal muscle. As is well-known, TNF-α triggers apoptosis by activating caspase-8 through TNF Complex IIa or IIb, while activated caspase-8 blocks necroptosis by inhibiting RIPK1 and RIPK3 [26, 43]. Noteworthy, we found that caspase-8 inhibitor Z-IETD-FMK alone could not reserve TNF-α-induced pyroptosis. One recent study demonstrated that activated RIPK1 can cleave GSDME to induce pyroptosis [44]. Hence, it is possible that activated RIPK1 due to the inhibition of caspase-8 cleaves GSDME and triggers pyroptosis in myotubes. Then, RIPK3 inhibitor GSK'872 was used to block necroptosis signaling pathway and TNF-α-induced pyroptosis in myotubes was completely reserved by Z-IETD-FMK in combination with GSK'872. Similar effect on reserving TNF-α-induced pyroptosis in myotubes was also found in the addition of caspase-3 inhibitor Z-DEVD-FMK. Our observations confirmed previous study on the role of TNF-α in triggering the crosstalk between apoptosis and pyroptosis [45]. Furthermore, Z-IETD-FMK together with GSK'872 reserved the reduced MHC1 in TNF-α-treated myotubes, while Z-DEVD-FMK also perform the same effect on the expression of MHC1 in TNF-α-treated myotubes. These further indicated that TNF-α may drive the loss of muscle fibers by triggering the crosstalk between apoptosis and pyroptosis.

We then investigated that knockdown of GSDME not only blocked the pyroptosis induced by TNF-α but also increased the expression of MHC1 reduced by TNF-α. These results further validated that TNF-α promotes sarcopenia by regulating GSDME-mediated pyroptosis. Notably, although knockdown of GSDME could reserve the expression of MHC1 reduced by TNF-α, it only partially reserved the expression of MHC1 as compared with untreated myotube, indicating that inhibition of pyroptosis may partially reserve the expression of MHC1 reduced by TNF-ɑ. One potential explanation is that although pyroptosis is inhibited, TNF-ɑ can still activate caspase-8/-3 and eventually stimulate apoptosis, which may also cause damage to the skeletal muscle. A recent study also found that knockdown of GSDME in macrophages reserved cell death from pyroptosis to apoptosis [46]. Hence, both activated apoptostic signaling and GSDME-mediated pyroptosis may co-participate in the development of sarcopenia.

Although our results suggested that inhibiting pyroptosis partially improves sarcopenia, we cannot completely negate the pathogenic role of GSDME-mediated pyroptosis in sarcopenia. Pyroptosis is a pro-inflammatory form of PCD that results in cell death and further tissue damage by inflammation cascades response [42]. Therefore, during the aging process, pyroptosis mediated by TNF-ɑ in skeletal muscle leads to, on the one hand, death of skeletal muscle fibers, on the other hand, skeletal muscle damage by releasing inflammatory factors. Furthermore, a recent study [47] demonstrated that GSDME-N cleaved by caspase-3 could also form pores in the mitochondrial membrane and promote the release of cytochrome-c to further activate caspase-3, resulting in a self-amplifying feedback loop that aggravated the damage to cells and tissues. Thus, GSDME might exacerbate the detrimental effects of pyroptosis on cells.

**Fig. 8:**
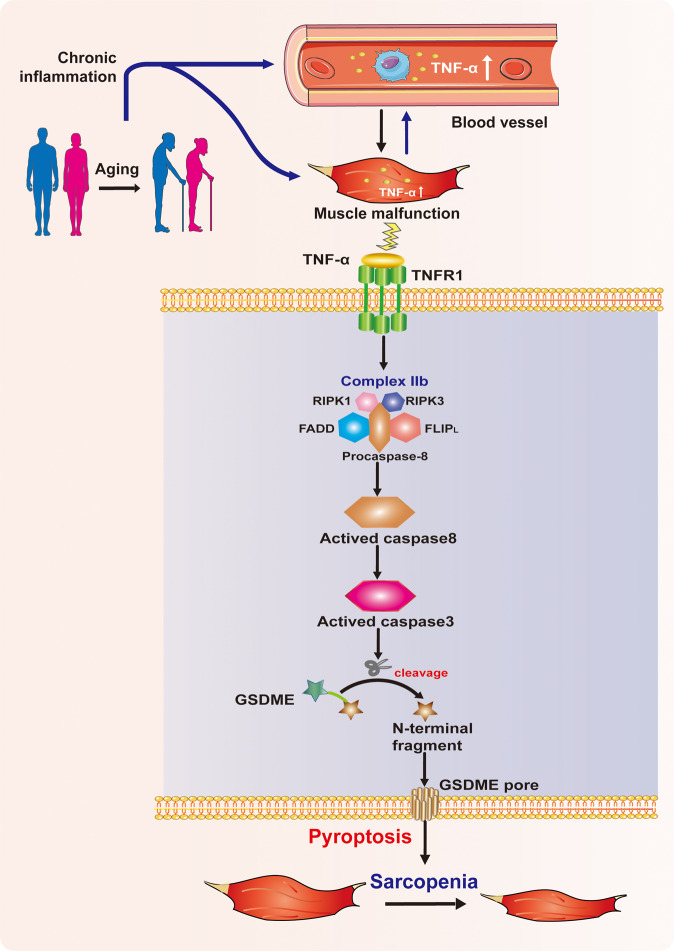
Model of TNF-α-induced sarcopenia by regulating caspase-8/caspase-3/GSDME-mediated pyroptosis through TNF Complex IIb. Aging leads to a chronic low-grade inflammation represented by the high level of serum TNF-α and local TNF-α overexpression in skeletal muscle. TNF-α binds to its receptor tumor necrosis factor receptor 1 (TNFR1) to assemble TNF Complex IIb on muscle cell surface. The assembly of TNF Complex IIb activates caspase-8 and downstream caspase-3. Activated caspase-3 further cleaves GSDME to release N-terminal fragment of GSDME. The intracytoplasmic N-terminal domain transfers to the membrane and forms pores in the cell membrane, executing pyroptosis in muscle cells. pyroptotic muscle cells result in loss of skeletal muscle fibers and finally lead to sarcopenia.

In summary, our study reveals a novel mechanism that TNF-ɑ/caspase-8/caspase-3/GSDME signaling-mediated pyroptosis plays a pathogenic role in the development of sarcopenia, as illustrated in Fig. 8. The molecular mechanism of pyroptosis and the crosstalk between apoptosis and pyroptosis in skeletal muscle cells might provide a more in-depth understanding of the pathogenesis of sarcopenia. It appears that caspase-3/GSDME signaling-mediated pyroptosis may be a promising therapeutic target for sarcopenia. There are still several limitations to our current study. Firstly, due to the long feeding time, we have not employed caspase-3 or GSDME knockout aged mice to validate the therapeutic significance of targeting caspase-3/GSDME-mediated pyroptosis. Secondly, we did not investigate the impact of inflammatory factors released by pyroptosis on the skeletal muscle of aged mice. Thirdly, we did not explore the original source of local increased TNF-α. As part of our group’s continuous efforts, we are now carrying out further studies on these issues.

## Materials and methods

### Antibodies and reagents

The following primary antibodies were used for immunoblotting, immunohistochemistry, and immunofluorescence: anti-GSDMD antibody (ab219800) and anti-GSDME antibody (ab215191) were purchased from Abcam (MA, USA). Anti-caspase-8 antibody (66093-1-Ig), anti-caspase-3 antibody (19677-1-AP), anti-TNF-α antibody(60291-1-Ig), anti-GAPDH antibody (60004-1-Ig), and anti-beta-Tubulin antibody (66240-1-Ig) were obtained from Proteintech (Wuhan, China). Cleaved-caspase-8 antibody (#8592) and anti-beta-actin antibody (#3700) was obtained from Cell Signaling Technology (MA, USA). Cleaved-caspase-3 antibody (GB11532) was purchased from Servicebio (Wuhan, China). Anti-myosin heavy chain 1 (MHC1) antibody was obtained from Developmental Studies Hybridoma Bank (DSHB, IA, USA). Alexa Fluor 594-labeled secondary antibody (ZF-0516) and Alexa Fluor 488-labeled secondary antibody (ZF-051) were purchased from Zhongshan Golden Bridge (Beijing, China). Mouse TNF-α ELISA Kit (EK282) was purchased from MULTISCIENCES (Hangzhou, China). DeadEnd™ Fluorometric terminal deoxynucleotidyl transferase-mediated digoxigenin-deoxyuridine nick-end labeling (TUNEL) System (G3250) was obtained from Promega (WI, USA). LDH cytotoxicity assay detection kit was obtained from Beyotime (Shanghai, China). Hoechst 33342/PI double stain kit was purchased from Solarbio (Beijing, China). Recombinant murine TNF-α was purchased from PeproTech (NJ, USA). Z-DEVD-FMK (A1920) and Z-IETD-FMK(B3232) were obtained from ApexBio Technology (TX, USA). Cycloheximide (CHX) and GSK’872 were obtained from MedChemExpress (NJ, USA). TGF-β-activated kinase 1 inhibitor (TAK1i) 5Z-7-Oxozeaenol was purchased from Sigma-Aldrich (MO, USA).

### Animal model and experimental design

Healthy male C57/BL 6 J mice were purchased from Shanghai SLACCAS Laboratory Animal Co., Ltd (certificate of quality SCXK 2017-0005), including 10 mice aged 12 weeks (weighting 24–26 g) as a young group, 10 mice aged 52 weeks (weighting 28–35 g) as a middle-aged group and 16 mice aged 80 weeks (weighing 30–40 g) as an old group. Mice were group-housed 4–5 per cage on a 12-hour light-dark cycle and bred under SPF conditions with suitable humidity and temperature for 56 days. Food and water were both available ad libitum. Body weight and grip strength were measured every 7 days. 1 mouse in the middle-aged group and 2 mice in the old group died during feeding. On day 56, there were 10 mice aged 20 weeks in the young group, 9 mice aged 60 weeks in the middle-aged group and 14 mice aged 88 weeks in the old group. All mice were fasted for 12 h overnight and euthanized for collecting blood and skeletal muscle samples at this time point. Blood samples were collected by retro-orbital venous plexus puncture for enzyme-linked immunosorbent assay (ELISA). Left and right GM were quickly isolated and weighted. The average mass of left and right GM was recorded as the absolute mass of GM. To exclude the influence of body weight on muscle mass, the absolute mass of GM was divided by body weight, which was defined as GMI. Right GM tissues were immediately frozen in liquid nitrogen and stored at −80 °C for western blot and gene expression studies. Left GM tissues were divided into two parts at the mid-belly. The proximal parts were fixed and stored in 4% parafomaldehyde for histological staining, whereas the distal parts were cut into pieces and fixed with 2.5% glutaraldehyde in 0.1 M phosphate buffer for TEM. The tubes containing the samples were digitally labeled to ensure examiners blinded. When establishment of sarcopenia model was confirmed,8 mice were randomly selected from the three groups respectively using random number table and used for the following experiments. All experiments were approved by the Animal Ethics Committee (Ethics Number: FJMU IACUC 2021-0385).

### Grip strength test

The grip strength of the four limbs of mouse was measured using mice grip strength dynamometer (YLS13A, Nuoleixinda, Tianjin, China). Mouse was gently placed on the mesh bar to allow its four limbs to hold the mesh. As the mouse grasped the mesh bar, its tail was gently pulled backward in a horizontal direction parallel to the mesh bar until the mouse released its grasp. The peak force applied by the mouse to grasp the mesh bar was recorded. Each mouse was tested repeatedly for six times. The average of six grip strength measurements was recorded as the absolute grip strength. To exclude the influence of body weight on muscle strength, the absolute grip strength was divided by body weight, which was defined as relative grip strength.

### Enzyme-linked immunosorbent assay

Blood samples were centrifuged at 3000 g for 20 min in a refrigerated centrifuge. Then serum was separated and stored at -80 °C. The concentrations of serum TNF-α were measured using a high sensitivity ELISA Kit following the supplier’s protocol. Data were analyzed using Excel.

### Histological analysis

Fixed GM tissues were dehydrated, embedded in paraffin, and sectioned at 10μm. Sections were dewaxed, and rehydrated for HE staining, immunofluorescence, immunohistochemistry and TUNEL staining. Sections were stained with hematoxylin and eosin for morphological evaluation and myofiber CSA analysis. Images were taken by Nikon Eclipse E200 microscope (Nikon, Japan). At least five random fields at adequate magnification were acquired for each section. The mean CSA of fibers (at least 250) in five fields for each section was analyzed using Image J software. Representative images were presented at 40x magnification. Immunofluorescence was performed to assess the expression and location of GSDME. After antigen retrieval with Tris-EDTA by using a microwave oven, sections were permeabilized 0.5% TritonX-100 in PBS for 20 min, then blocked with 5% BSA in PBS for 1 h. Following blocking treatment, sections were incubated overnight at 4 °C with primary antibody against GSDME (1:50). Alexa Fluor 594-labeled secondary antibody (1:200) was used as a detection antibody. Nuclei were labeled with DAPI (Cell Signaling Technology, USA, 1 μg/ml). Confocal images were acquired by using Zeiss confocal laser scanning microscope (LSM800, Zeiss, Germany). Immunohistochemistry was performed to assess the expression of cleaved-caspase-8 and cleaved-caspase-3. After antigen retrieval, sections were incubated with 3% hydrogen peroxide to inactive endogenous peroxidases and blocked with 10% bovine serum albumin. Following blocking treatment, sections were incubated overnight at 4 °C with primary antibody (cleaved-caspase-8 rabbit monoclonal antibody, diluted 1:3000; cleaved-caspase-3 rabbit polyclonal antibody, diluted 1:1000). Sections were incubated with secondary antibodies, developed with DAB and counterstained with hematoxylin. Stained sections were photographed by using Nikon Eclipse E200 microscope (Nikon, Japan). Images were analyzed by using Image-Pro Plus software. To analyze cell death, sections were stained with TUNEL according to the manufacturer’s instructions. TUNEL-positive cells were counted on five random fields of each section (20x magnification) using Image J software.

### Transmission electron microscope

For detecting ultrastructure of GM tissues, tissues were cut into pieces approximately 1 mm^3^ in size and fixed with 2.5% glutaraldehyde in 0.1 M phosphate buffer. For detecting ultrastructure of cells, cells were fixed with 2.5% glutaraldehyde in 0.1 M phosphate buffer at room temperature for 5 min and then gently scraped, centrifuged and postfixed in 1%OsO4.Tissues and cells were embedded in resin for ultrathin sectioning. Ultrathin sections were then stained with uranyl acetate and lead citrate. Images were acquired by using Transmission electron microscopy (HT7800, HITACHI, China).

### Cell culture, differentiation and treatment

Mouse C2C12 myoblast cell line (#CL-0044) was kindly provided by Procell Life Science&Technology Co., Ltd (Wuhan, China). Myoblasts were cultured in high-glucose Dulbecco’s Modified Eagle’s Medium (DMEM, BasalMedia Technologies Co., LTD, Shanghai, China) supplemented 10% fetal bovine serum (FBS, ExCell Bio, Guangzhou, China) and 1% penicillin-streptomycin (Meilunbio, Dalian, China) at 37 °C in a humidified atmosphere with 5% CO_2_. To induce myoblasts differentiation into myotubes, the medium was replaced with DMEM supplemented with 2% horse serum (Procell, Wuhan, China) when the cells were >90% confluent. The differentiated medium was changed daily for 5 days. Differentiated myotubes were identified with immunofluorescence staining. Thereafter, Myotubes were either treated or not treated with drugs, depending on the experimental condition.

C2C12 myoblasts were seeded in 6-well or 12-well plate, differentiated into myotubes and starved in serum-free medium for 24 h before the stimulation. For TNF-α treatments, the culture medium was switched to fresh medium containing different concentrations (0,1,10,100 ng/ml) of TNF-α for a period as indicated in the figures. For TNFα + CHX or TNF-α + TAK1i treatments, myotubes were treated with either 100 ng/ml TNF-α plus 10 μg/mL CHX to trigger TNF Complex IIa or 100 ng/ml TNF-α plus 1 μM TAK1 inhibitor 5z-7-oxozeaenol to trigger TNF Complex IIb for the indicated time points. To inhibit caspase-8 activity, myotubes were pre-incubated for 30 min with 1 μM GSK'872 and 1 h with 50 μM Z-IETD-FMK prior to treatment with 100 ng/ml TNF-α for 72 h.To inhibit caspase-3 activity, myotubes were pre-incubated for 1 h with 50 μM Z-DEVD-FMK prior to treatment with 100 ng/ml TNF-α for 72 h.

### Immunofluorescence staining

Myoblasts were seeded on sterile coverslips and induced to differentiate into myotubes. Myotubes were fixed with 4% paraformaldehyde for 15 min at room temperature, permeabilized with 0.25% TritonX-100 for 10 min at 4 °C and blocked with 5% BSA for 30 min at room temperature. Then myotubes were incubated with anti-MHC1 (1:100 dilution) antibody overnight at 4 °C. Following primary antibody incubation, myotubes were washed with PBS and incubated with an Alexa Fluor 488-labeled secondary antibody (1:200 dilution) for 1 h, and counterstained with DAPI (Cell Signaling Technology, USA, 1 μg/ml). Fluorescence images were taken by using Nikon Eclipse TS2R microscope (Nikon, Japan).

### Western blot

For detecting the protein expression in GM tissues, approximately 25 mg of tissues were dissected and homogenized in 500 μl RIPA lysis buffer (Beyotime, Shanghai, China) with a Mill Cryogenic Grinder (KZ-III-FP, Servicebio Technology, Wuhan, China). For detecting the protein expression in cultured cells, cells and supernatants were both collected for protein extract after treatments and then extracted using lysis buffer (Beyotime, Shanghai, China) containing 1 mM PMSF (Beyotime, Shanghai, China) and 1% protease inhibitor cocktail (Roche, Switzerland). After protein concentrations were detected by BCA Protein Assay (Beyotime, Shanghai, China), western blot analysis was performed as described previously [48]. Briefly, the protein lysates were separated on sodium dodecyl sulfate-polyacrylamide gel electrophoresis (7.5%, 12.5%, or 15%) and electroblotted onto PVDF membranes. The PVDF membranes were blocked with 5% skimmed milk and then incubated with primary antibodies overnight at 4 °C. Anti-GSDMD antibody and anti-GSDME antibody were diluted at 1:1000, anti-caspase-8 antibody and anti-caspase-3 antibody were diluted at 1:500, anti-MHC1 antibody was diluted at 1:5000, anti-GAPDH, anti-β-Tubulin, and anti-β-actin were diluted at 1:2000. Following primary antibody incubation, membranes were washed with 1× tris-buffered saline with Tween and incubated with appropriate secondary antibodies. Finally, membranes were detected using an ECL kit (Beyotime, Shanghai, China). Relative intensities of the protein bands were analyzed using Image J software.

### LDH release assay

Myoblasts were seeded on 6- or 12-well plates, induced to differentiate into myotubes, and treated as indicated. Cell death was quantitated by assaying LDH release into cell culture supernatants using LDH cytotoxicity assay detection kit according to the manufacturer’s protocol. Briefly, Culture supernatants were harvested and centrifuged at 400 × *g* for 5 min after various treatments. Aliquots of supernatants were transferred into 96-well plates, subjected to the LDH working liquids, and incubated for 30 min at room temperature protected from light. Thereafter, the absorbance was measured at 490 nm. The percentage of LDH release was calculated as follows: %release = 100 × (experimental LDH release—spontaneous LDH release)/ (maximal LDH release—spontaneous LDH release). Duplicate wells were run in each experiment, and each experiment was repeated four or five times.

### Hoechst 33342/ PI double staining assay

Myoblasts were seeded on 6- or 12-well plates, induced to differentiate into myotubes, and treated as indicated. Cell death was quantitated by assaying Hoechst 33342/PI double staining using Hoechst 33342/PI double stain kit according to the manufacturer’s protocol. The medium of each well was replaced with 1 ml of staining buffer containing 5 µl of Hoechst 33342 and 5 µl of PI, followed by incubation for 30 min at 4 °C without light. Thereafter, cells were washed with PBS and observed using Nikon Eclipse TS2R microscope (Nikon, Japan). Four fields per well were randomly selected for counting the stained cells by Image J software.

### Stable GSDME knockdown cell lines construction and differentiation

To construct cell lines with stable knockdown GSDME, a GSDME knockdown lentivirus (LV-shGSDME) was designed and constructed by Zolgene Biotech (Fuzhou, China). For LV-shGSDME, the shRNA sequence of GSDME, 5’-GGA GCT GTT TGT GAA ACA AGA-3’, was cloned into the hU6-MCS-CMV-ZsGreen-PGK-Puromycin vector. The empty lentiviral vector was used as a negative control (LV-shNC). C2C12 myoblasts were transfected with LV-shGSDME at an optimal MOI determined in a preliminary experiment. After incubation for 72 h, myoblasts were selected with 4 μg/mL puromycin to construct stable knockdown myoblasts. Knockdown efficiency was confirmed by western blot. Thereafter, the stable GSDME knockdown myoblasts were induced to differentiate into myotubes by replacing DMEM supplemented with 2% horse serum. After 5 days for differentiation, the expression of MHC1 protein was detected by western blot to identify myotubes.

### Statistical analysis

Data are presented as the mean ± standard deviation (SD) and analyzed with Graph Prism 9.0 software. Comparisons among groups were performed using one-way analysis of variance (ANOVA). Pairwise comparisons of multiple groups were performed by SNK-*q* test. Pearson correlation analysis was used for simple linear correlation analysis. All the experiments were repeated at least four times, and a value of *P* < 0.05 was considered statistically significant.

## Supplementary information

supplemental figure legends

supplemental figure

Original western blots

## Data Availability

All data generated and analyzed during this study are included in this article and its Supplementary Information files. All data are available from the corresponding author upon reasonable request.
