# Supplementary material for: TNF-α contributes to sarcopenia through caspase-8/caspase-3/GSDME-mediated pyroptosis
Source: Cell Death Discov. 2023 Feb 24;9:76. doi: 10.1038/s41420-023-01365-6 (PMC9950087; doi:10.1038/s41420-023-01365-6)
Supplement: Supplementary file 1 — supplemental figure legends [file 41420_2023_1365_MOESM1_ESM.docx]

**Supplemental figure, Determination of optimal MOI for infecting myoblasts with GSDME-silencing lentiviral vector and identification of differentiated myotubes transfected with lentivirus.**

A, Fluorescence images of myoblasts infected with GSDME-silencing lentiviral vector with different MOI values (0, 1, 5, 10, 50, 100) were observed by fluorescence microscopy. Scale bar:100μm. B, Immunoblot of GSDME in myoblasts infected with silenced GSDME lentivirus at different MOI values (0, 1, 5, 10, 50, 100). C, Immunoblot of MHC1 in lentivirus-transfected myoblasts and myotubes.
