# Supplementary figures and images for: TNF-α contributes to sarcopenia through caspase-8/caspase-3/GSDME-mediated pyroptosis
Source: Cell Death Discov. 2023 Feb 24;9:76. doi: 10.1038/s41420-023-01365-6 (PMC9950087; doi:10.1038/s41420-023-01365-6)

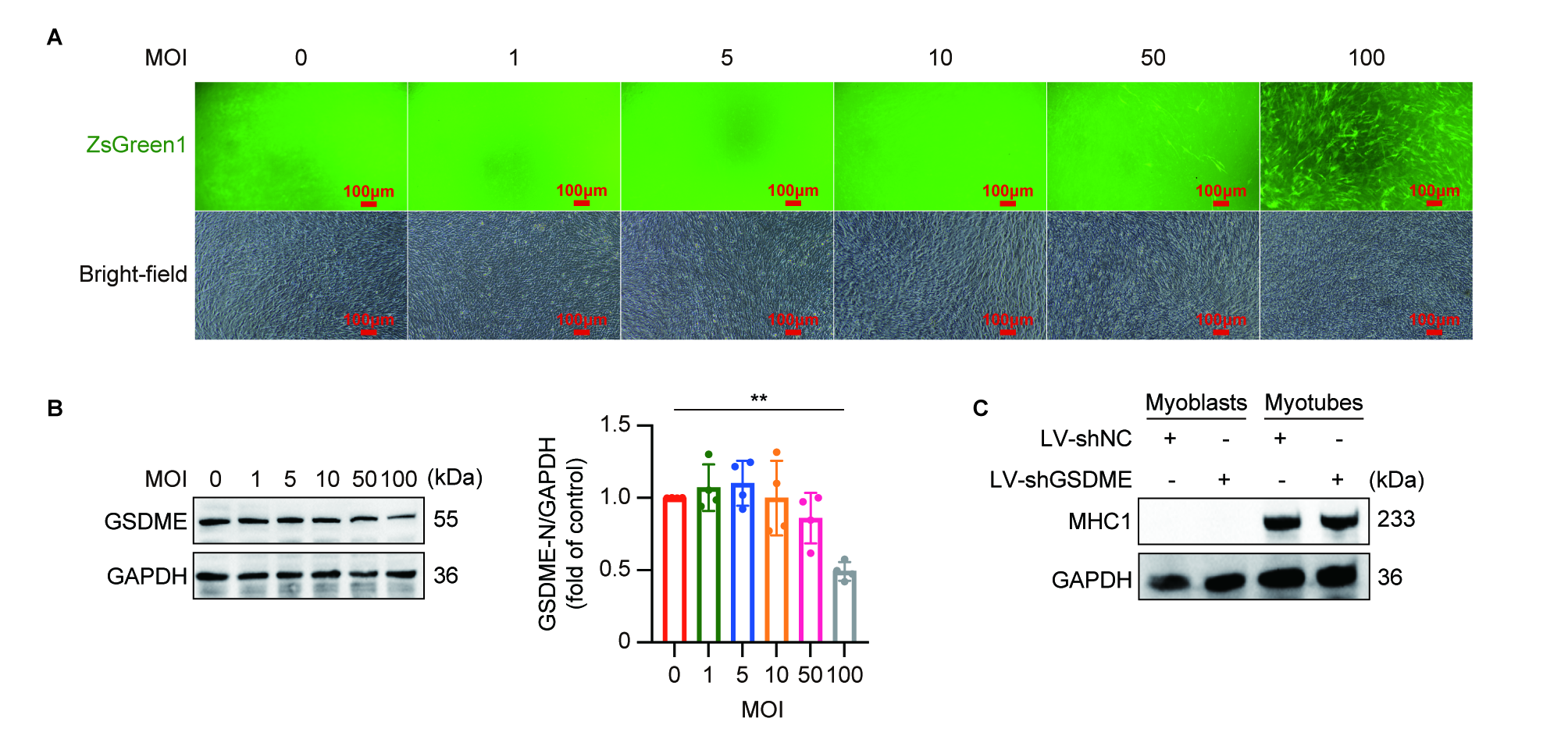

Supplement: Supplementary file 2 — supplemental figure [file 41420_2023_1365_MOESM2_ESM.tif]
