# Supplementary material for: TNF-α contributes to sarcopenia through caspase-8/caspase-3/GSDME-mediated pyroptosis
Source: Cell Death Discov. 2023 Feb 24;9:76. doi: 10.1038/s41420-023-01365-6 (PMC9950087; doi:10.1038/s41420-023-01365-6)

# Original western blots

Figure 1G
immunoblot of TNF-α

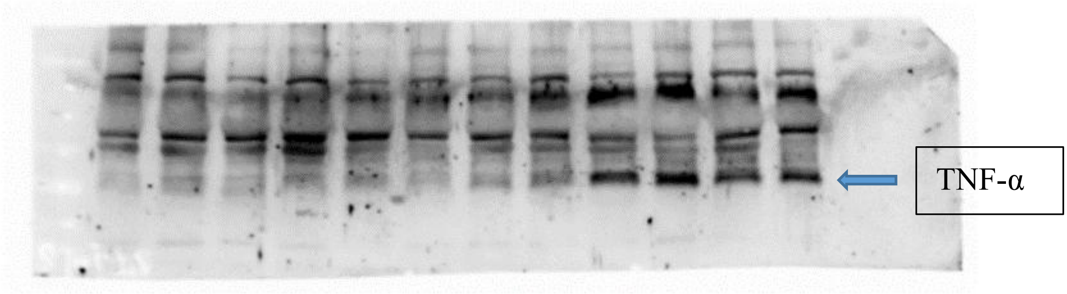


immunoblot of GAPDH

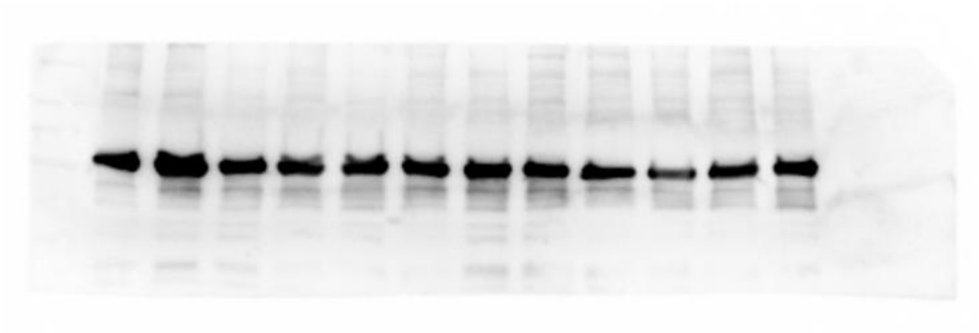

Figure2A
immunoblot of GSDMD and GSDMD-N

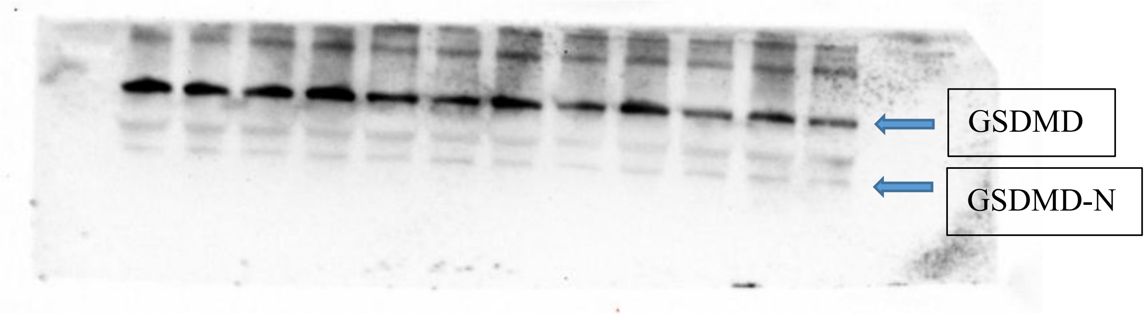


immunoblots of GAPDH of GSDMD

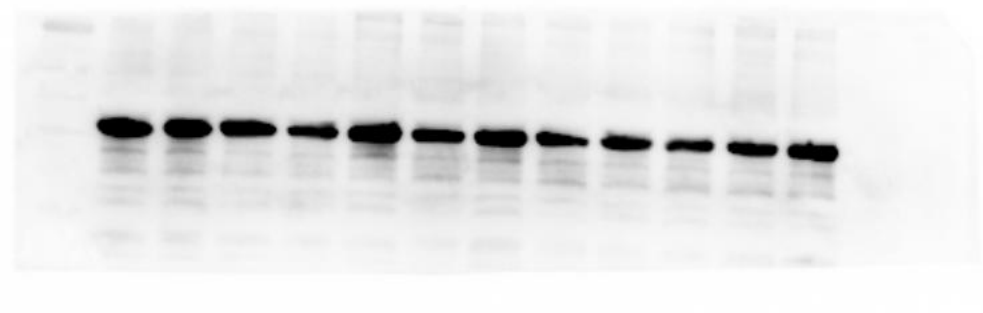

immunoblot of GSDME and GSDME-N

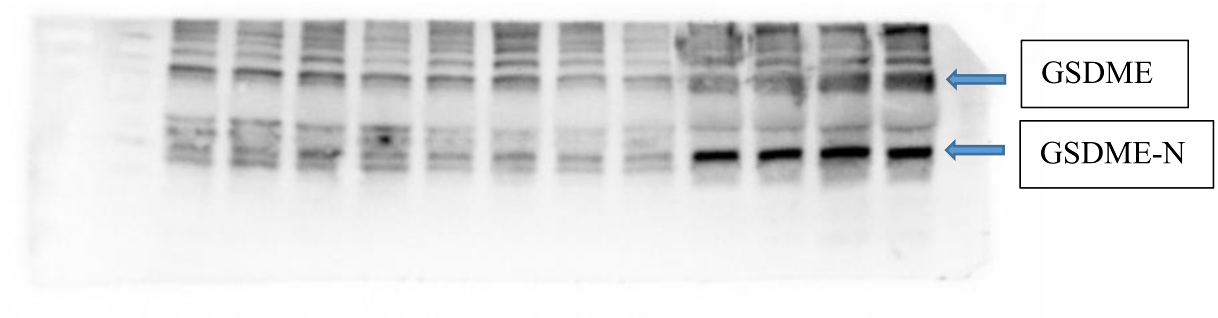


immunoblot of GAPDH of GSDME

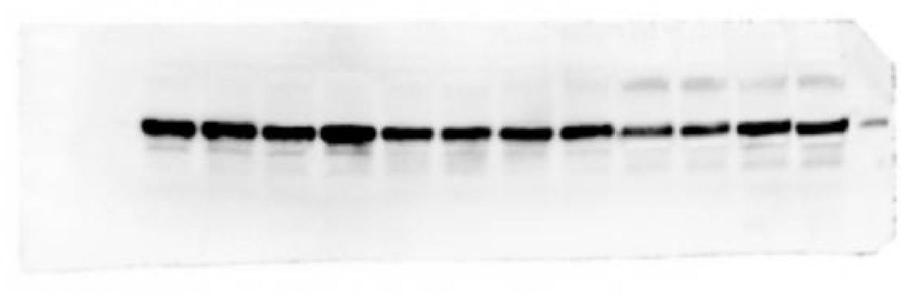

immunoblot of caspase-8 and cleaved-caspase-8

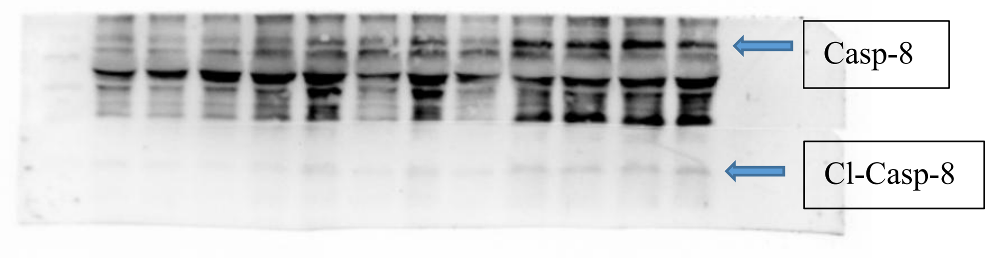


immunoblot of cleaved-caspase-8

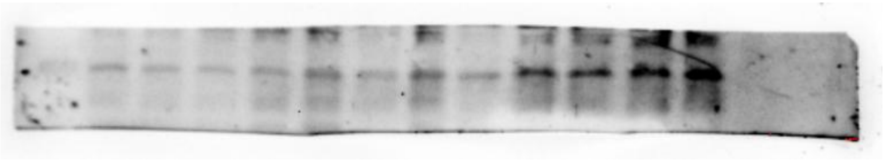

immunoblot of GAPDH of caspase-8

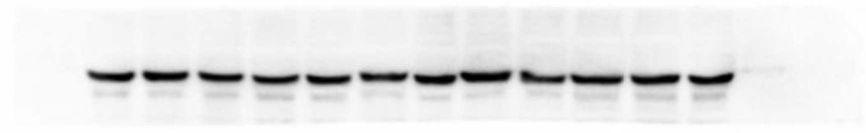

immunoblot of caspase-3 and cleaved-caspase-3

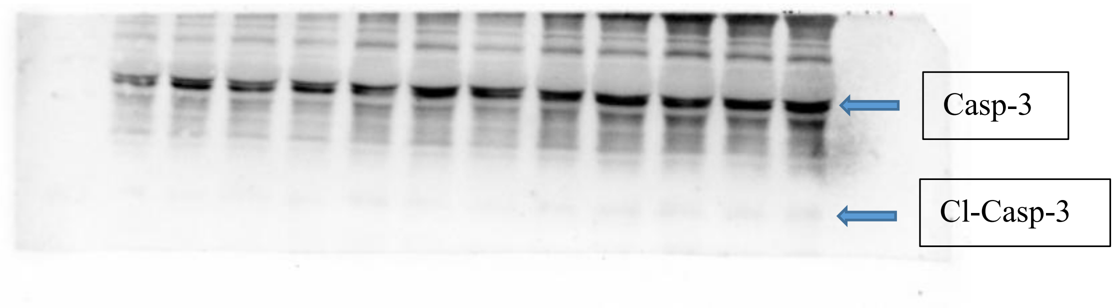


immunoblot of cleaved-caspase-3

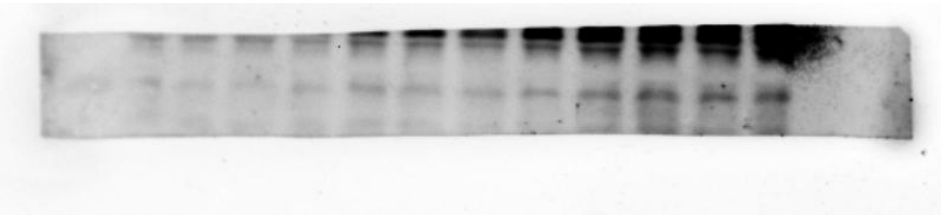

immunoblot of GAPDH of caspase-3

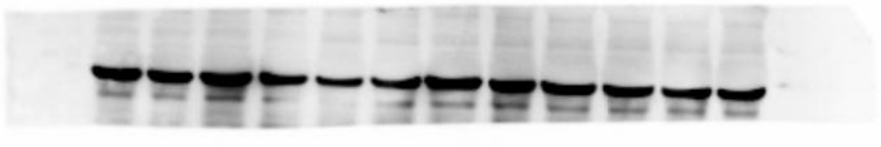

Figure3E
immunoblot of GSDME and GSDME-N

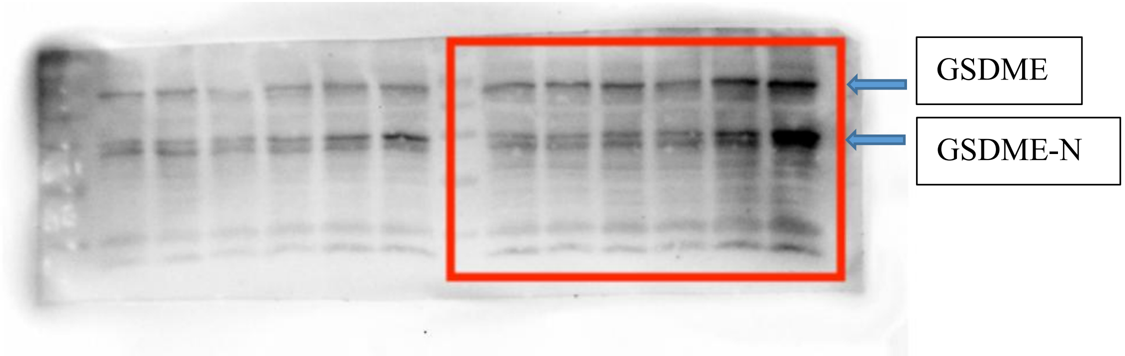


immunoblot of β-actin

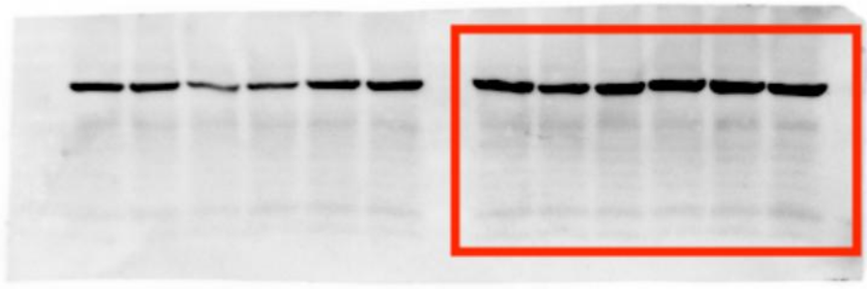

Figure 3F

immunoblot of GSDME and GSDME-N

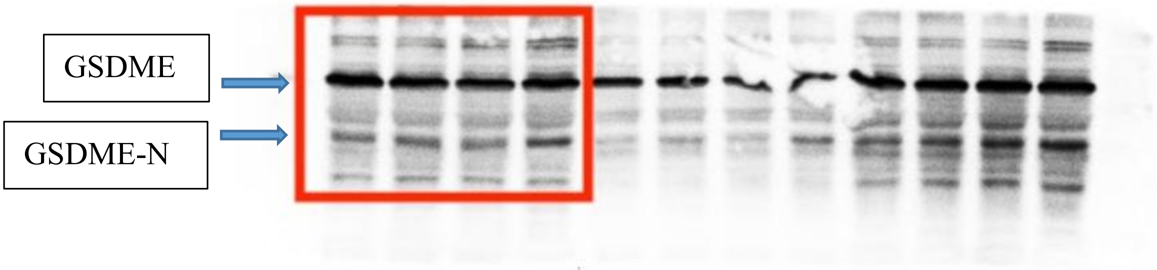


immunoblot of β-actin

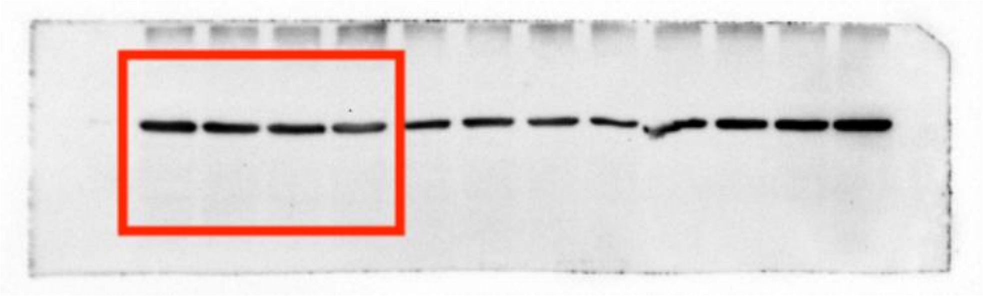

Figure 3G

immunoblot of GSDME and GSDME-N

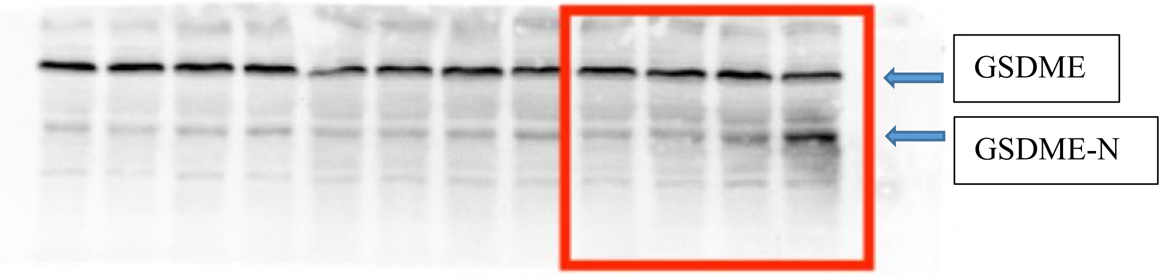


immunoblot of β-actin

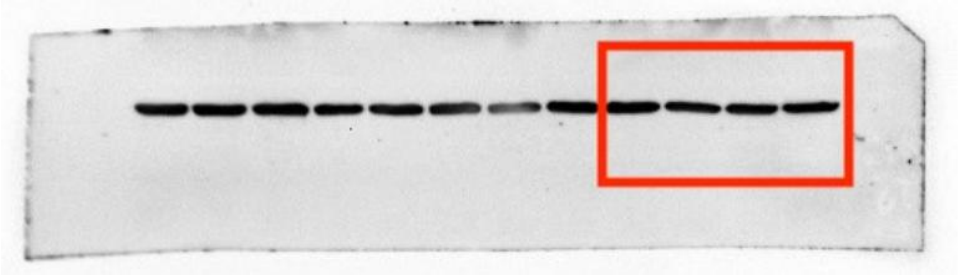

Figure 4A

immunoblot of caspase-8 and cleaved-caspase-8

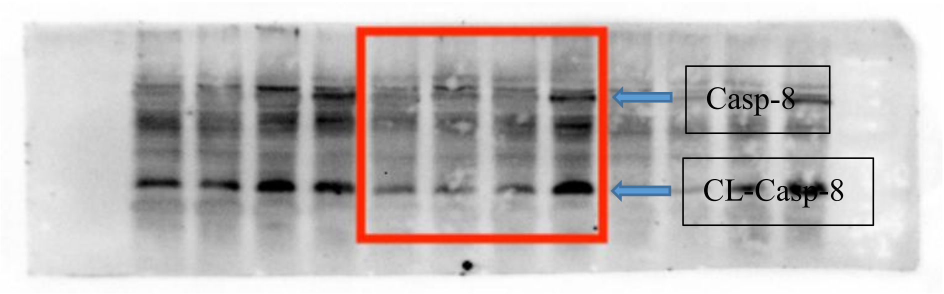


immunoblot of β-Tubulin

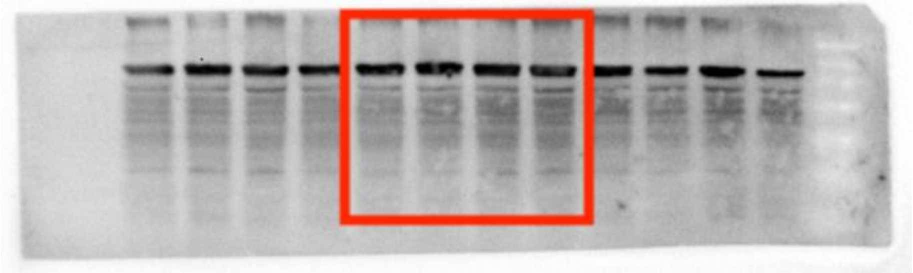

Figure 4B

immunoblot of GSDME and GSDME-N

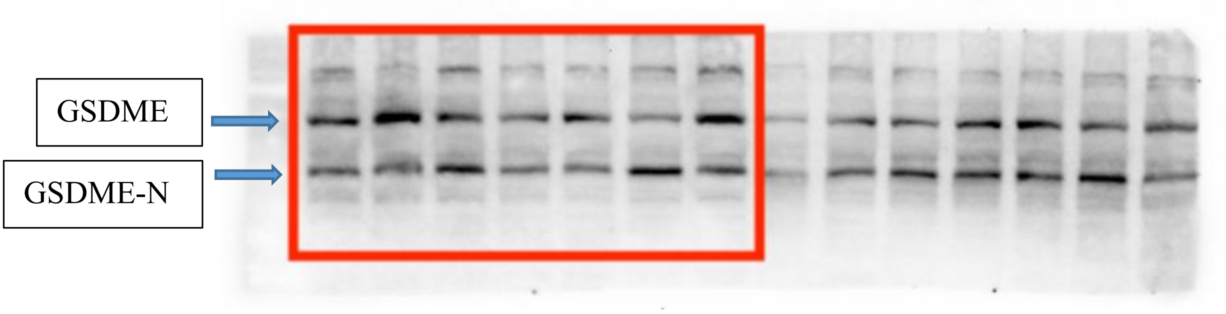


immunoblot of GAPDH of GSDME

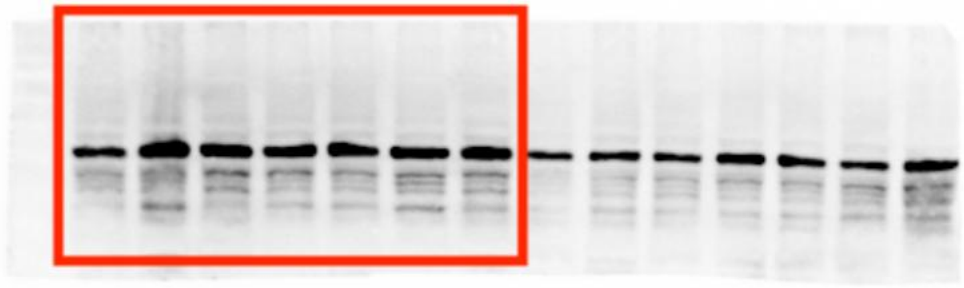

immunoblot of caspase-3 and cleaved-caspase-3

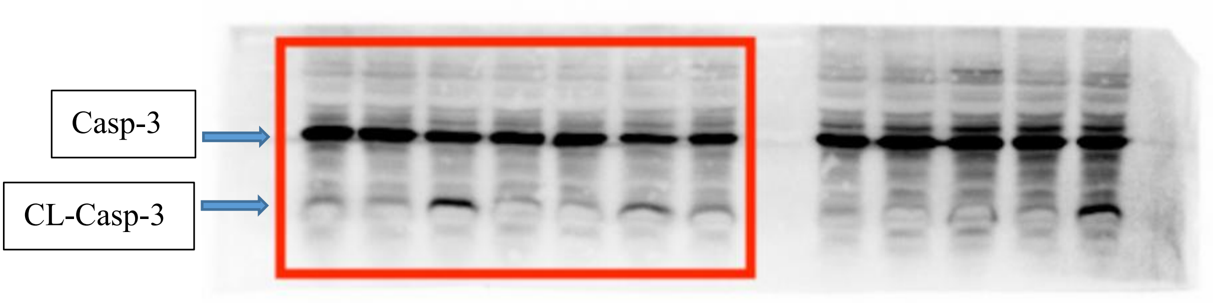


immunoblot of GAPDH of caspase-3

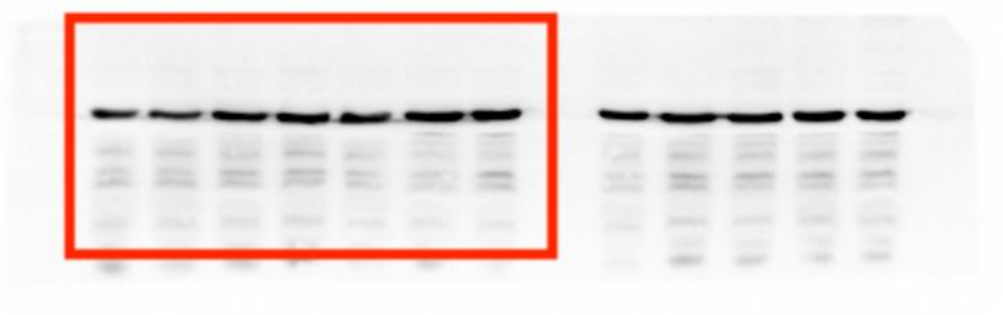

Figure 5A
immunoblot of caspase-3 and cleaved-caspase-3

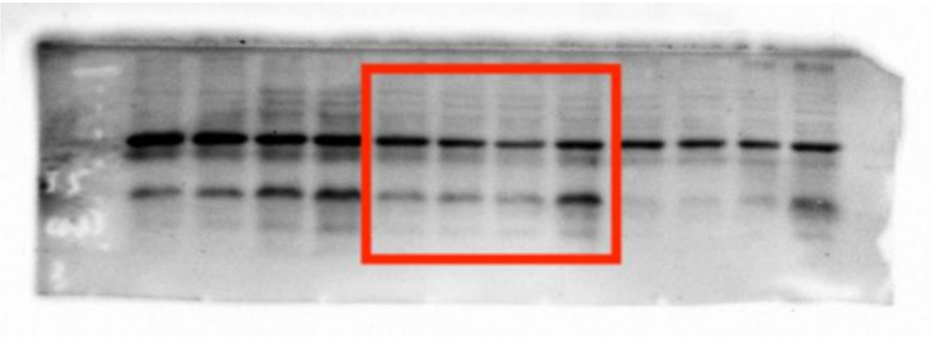

immunoblot of β-Tubulin

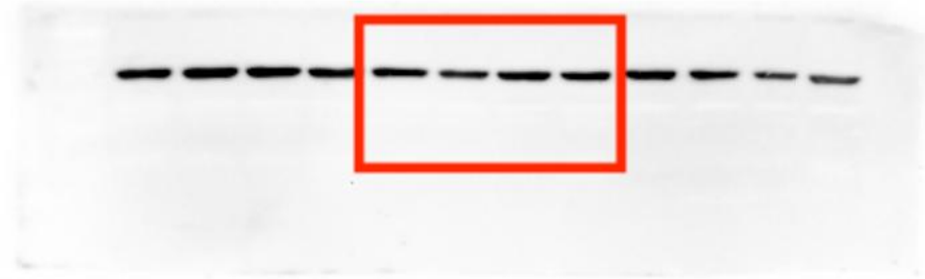

Figure 5B

immunoblot of GSDME and GSDME-N

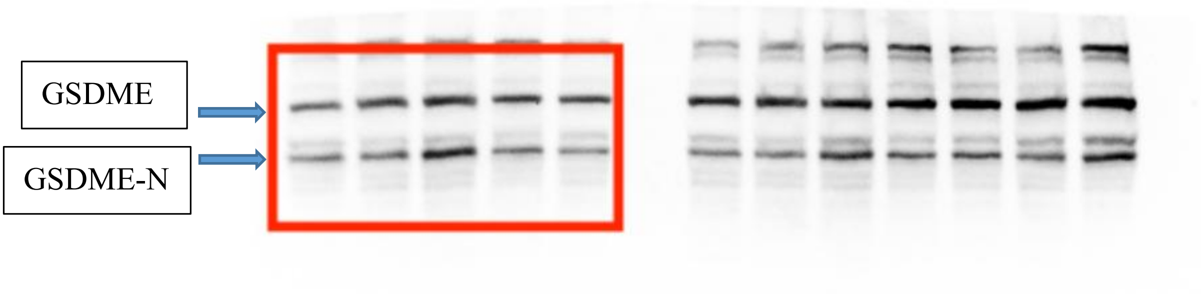


immunoblot of GAPDH of GSDME

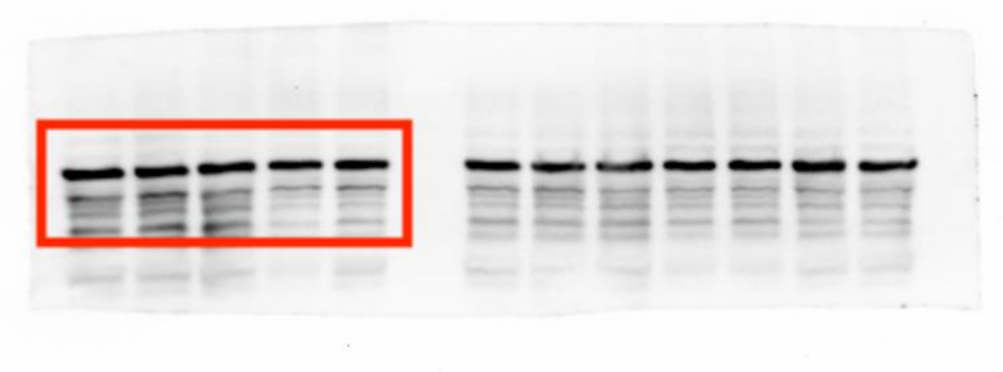

Figure 6A

immunoblot of GSDME and GSDME-N

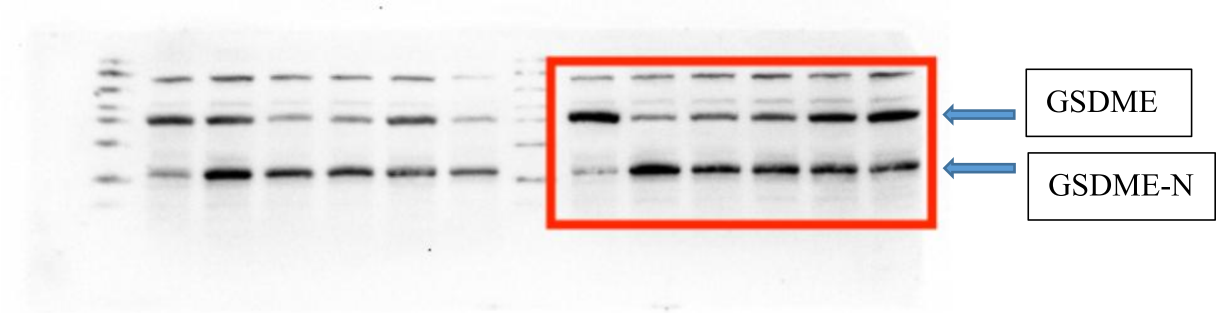


immunoblot of GAPDH

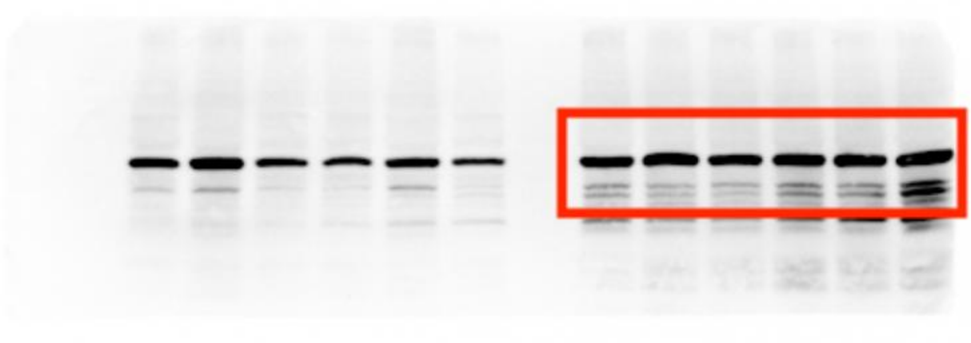

Figure 6B

immunoblot of GSDME and GSDME-N

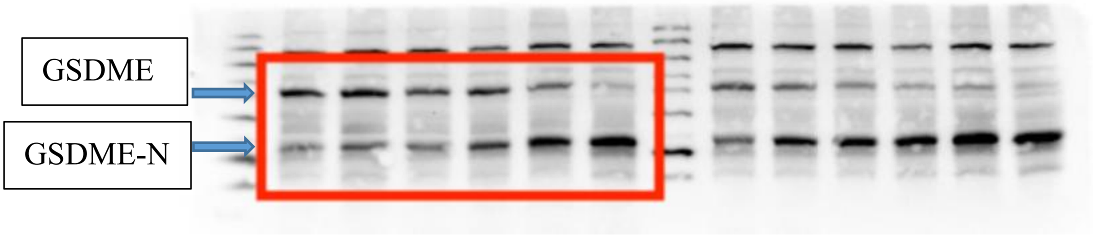


immunoblot of GAPDH

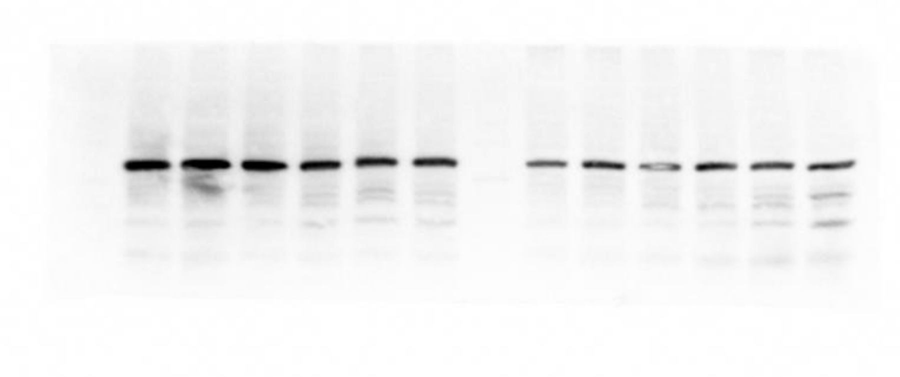

Figure 6C

immunoblot of GSDME and GSDME-N

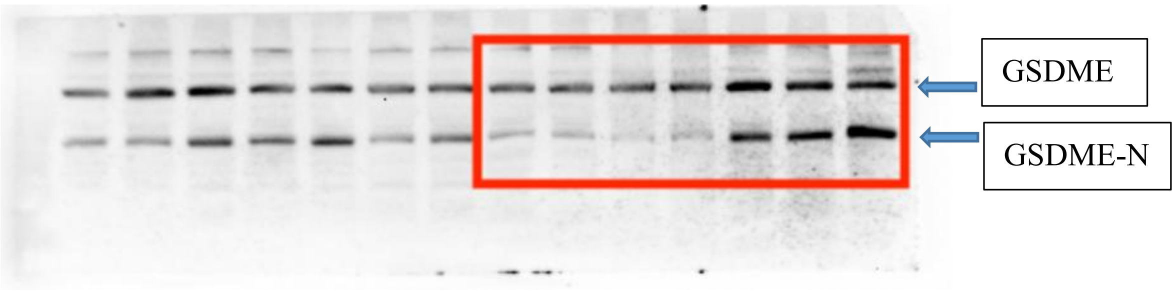


immunoblot of GAPDH

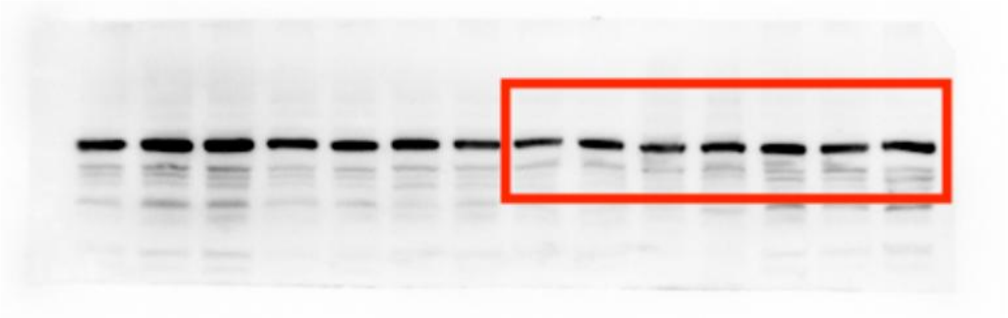

Figure 6D

immunoblot of GSDME and GSDME-N

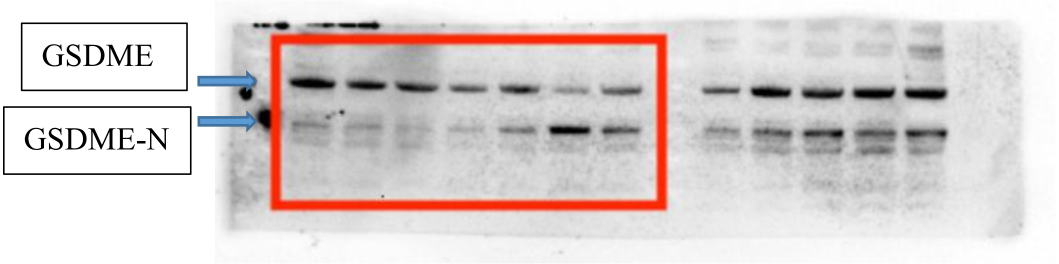


immunoblot of GAPDH of GSDME

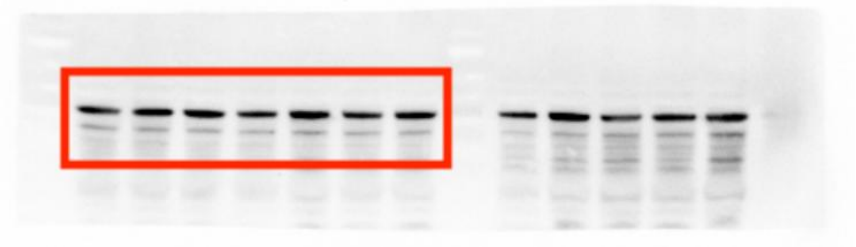

immunoblot of caspase-8 and cleaved-caspase-8

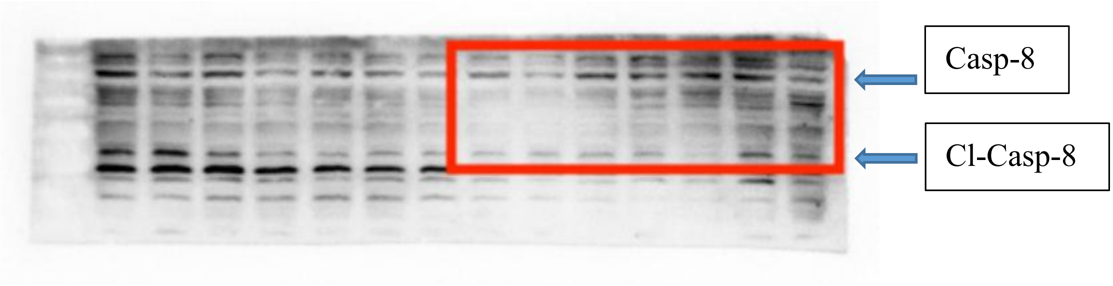


immunoblot of GAPDH of caspase-8

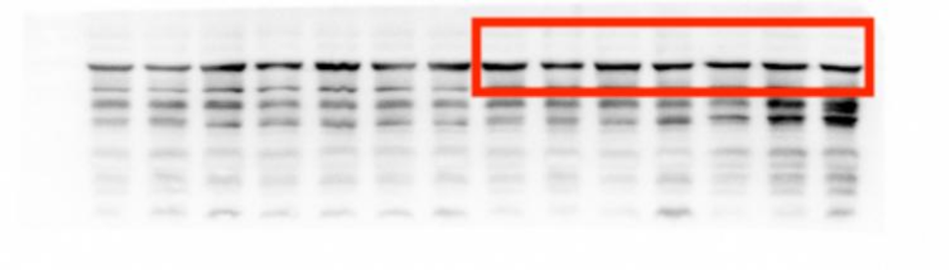

Figure 6F

immunoblot of GSDME and GSDME-N

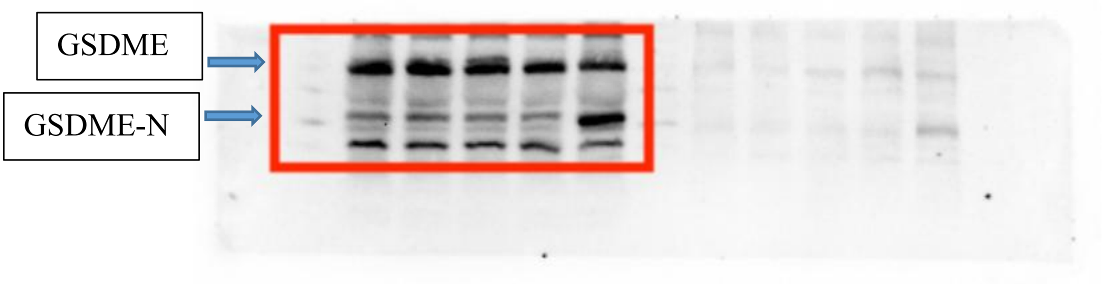


immunoblot of GAPDH of GSDME

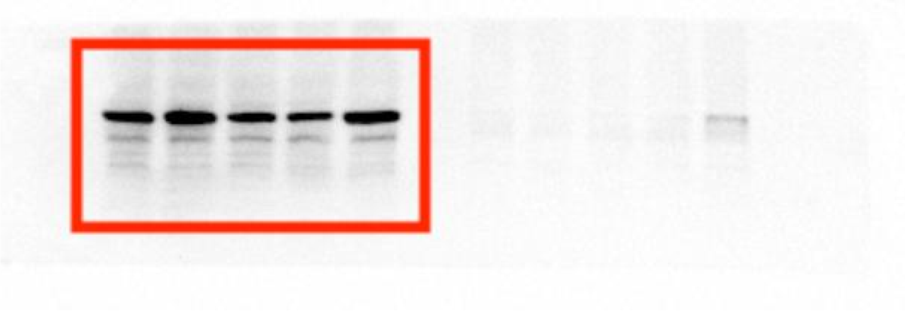

immunoblot of caspase-3 and cleaved-caspase-3

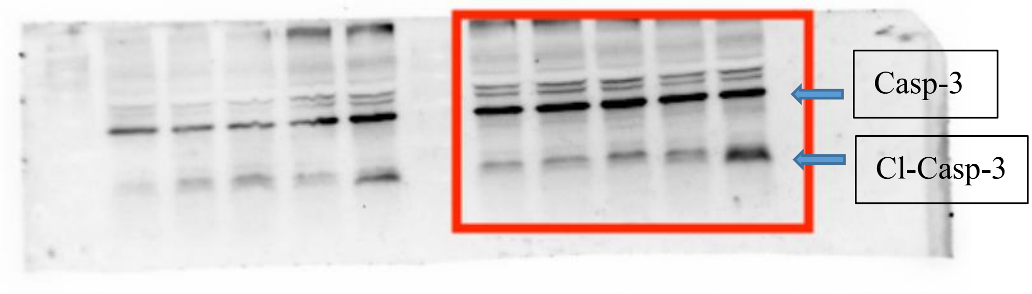


immunoblot of GAPDH of caspase-3

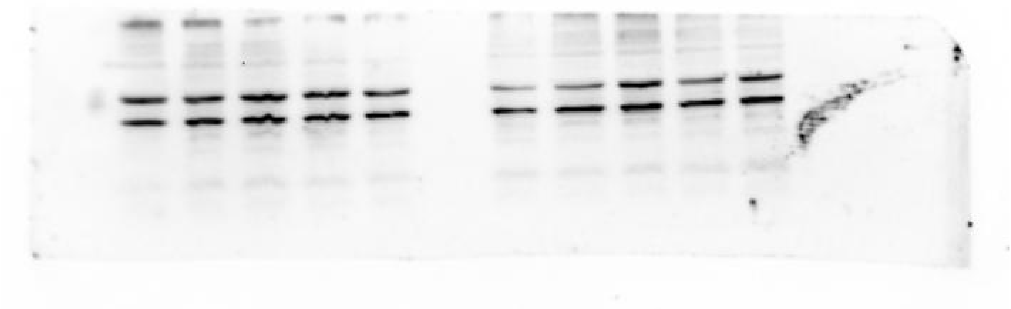

Figure 7A

immunoblot of MHC1

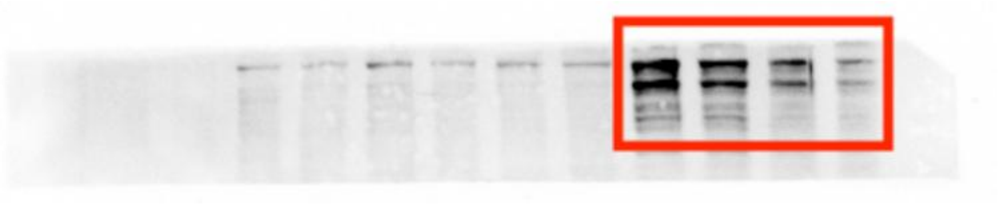

immunoblot of β-Tubulin of MHC1

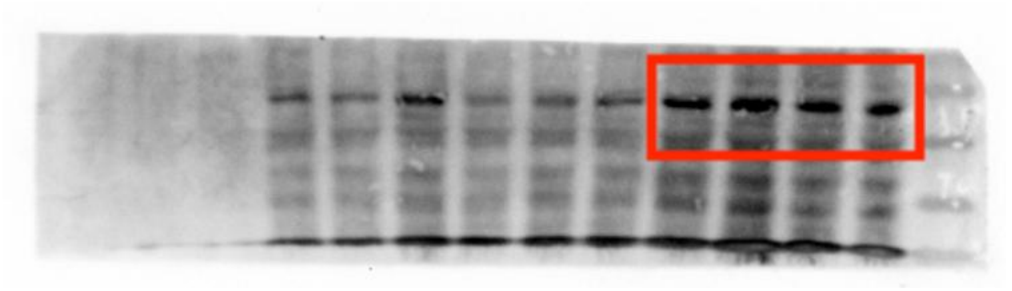

Figure 7B

immunoblot of MHC1

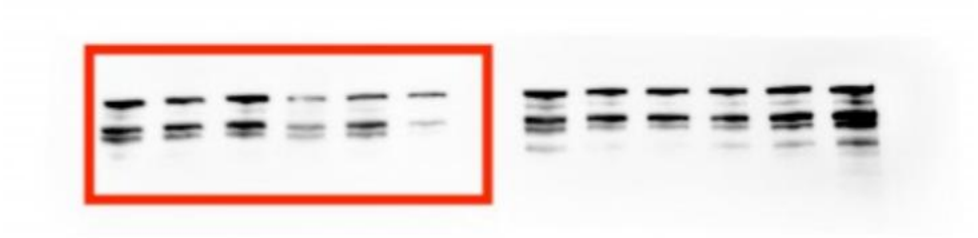

immunoblot of MHC1 and GAPDH

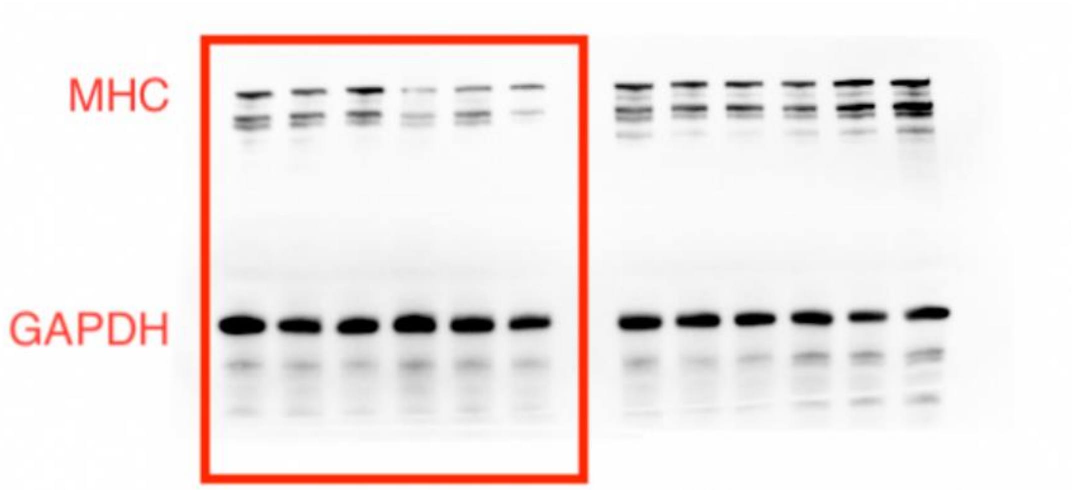

Figure 7C
immunoblot of MHC1

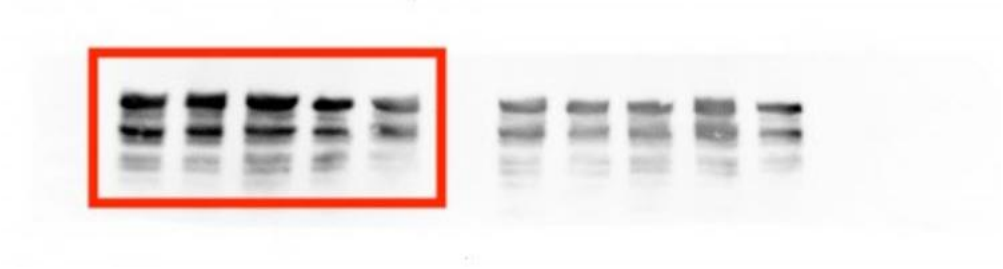

immunoblot of MHC1 and GAPDH

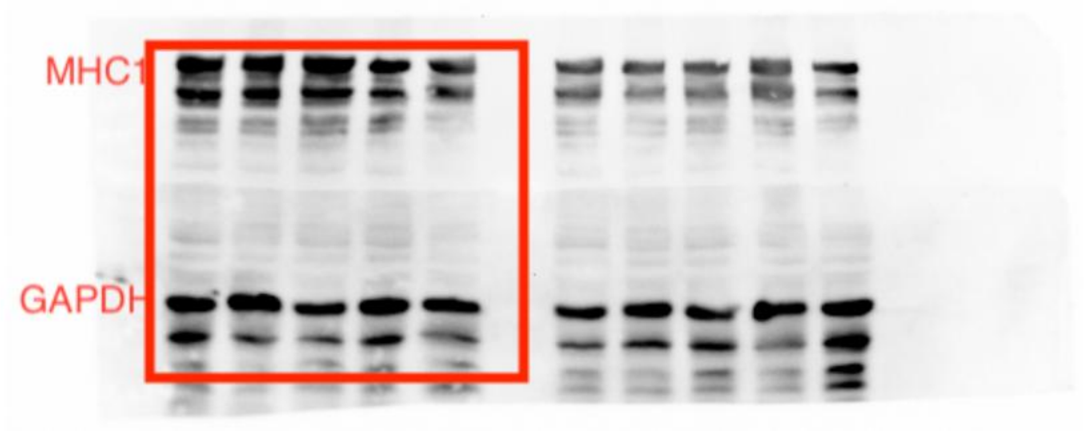


Figure 7D
immunoblot of MHC1

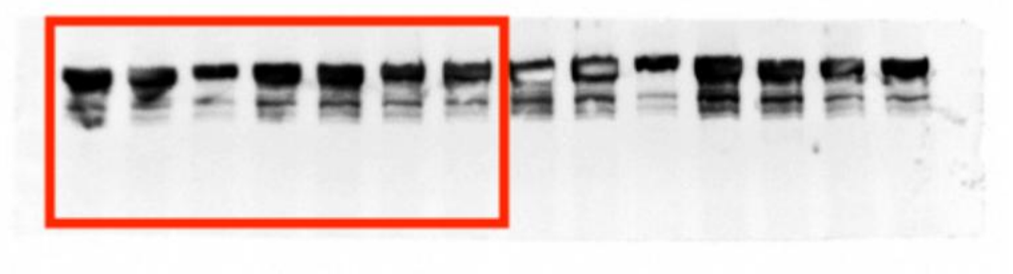

immunoblot of GAPDH

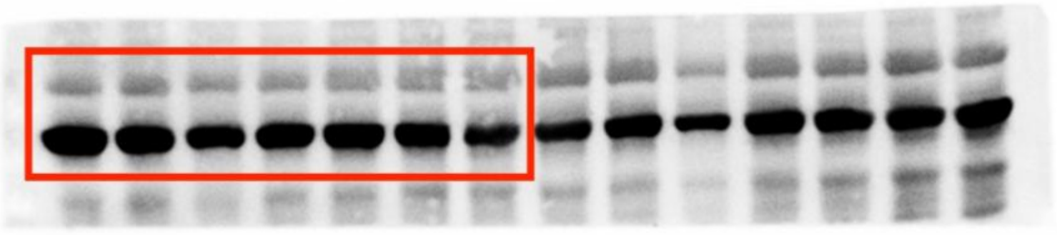

Figure 7E
immunoblot of MHC1

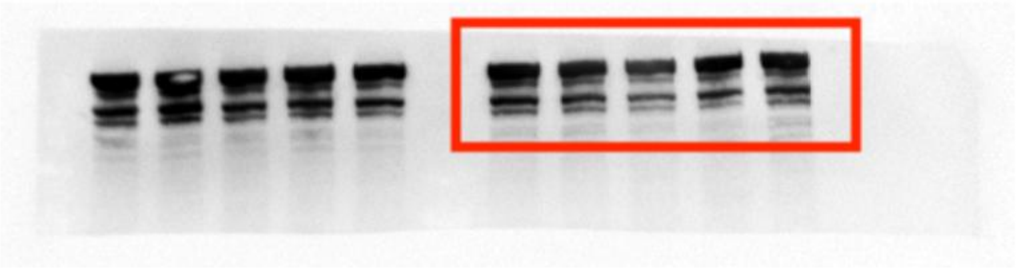

immunoblot of GAPDH

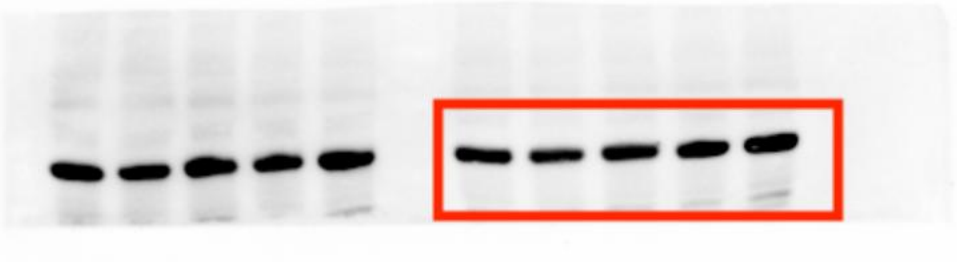

Figure 7F
immunoblot of GSDME

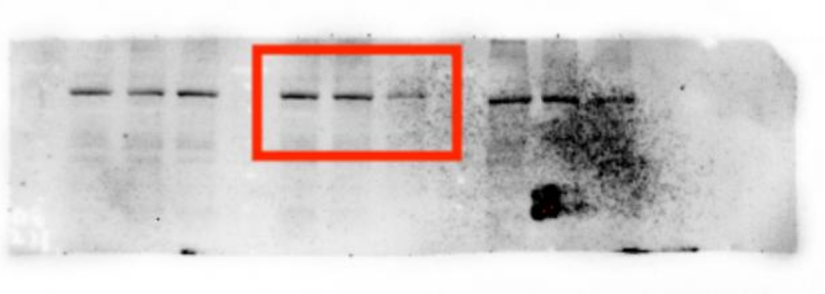

immunoblot of GAPDH

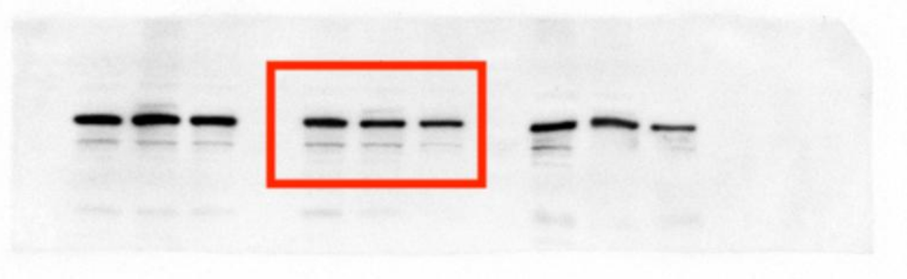

Figure 7H
immunoblot of GSDME

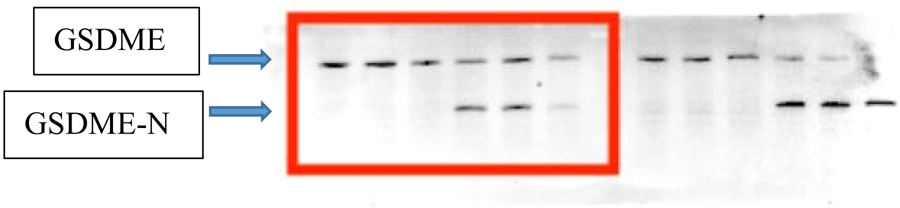


immunoblot of GAPDH

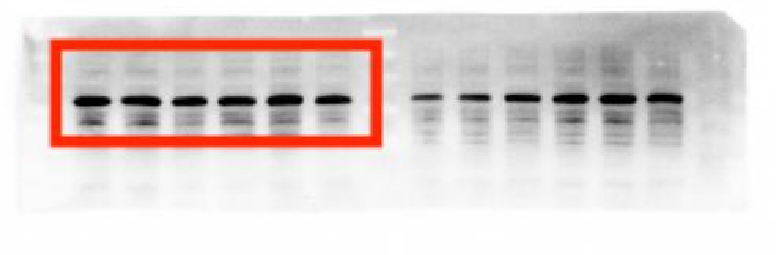

Figure 7I
immunoblot of MHC1

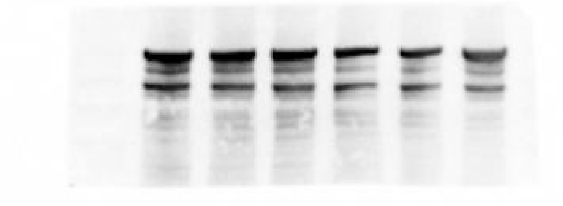

immunoblot of GAPDH

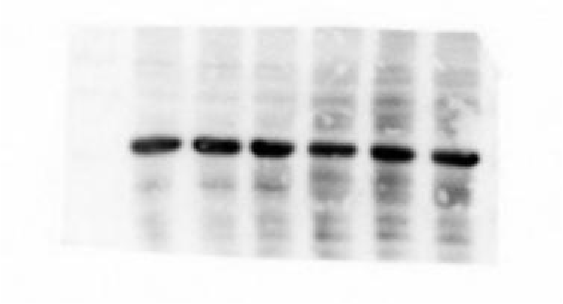

Supplemental figure B
immunoblot of GSDME

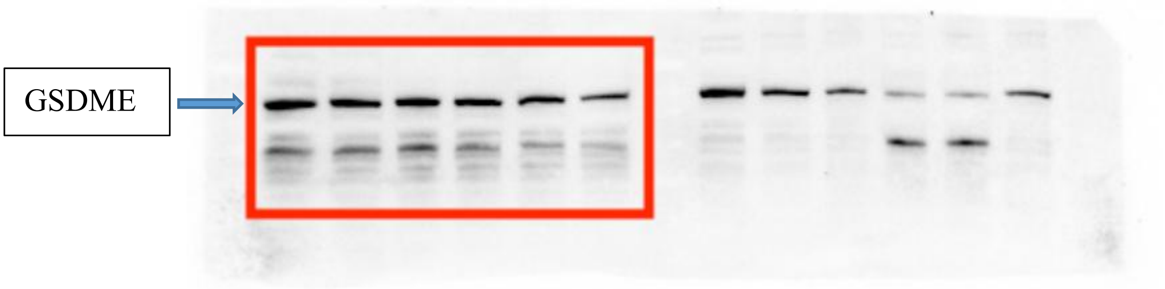


immunoblot of GAPDH

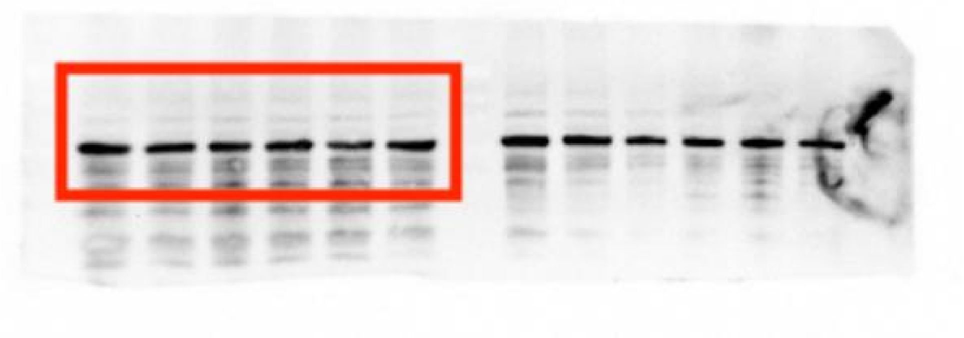

Supplemental figure C
immunoblot of MHC1

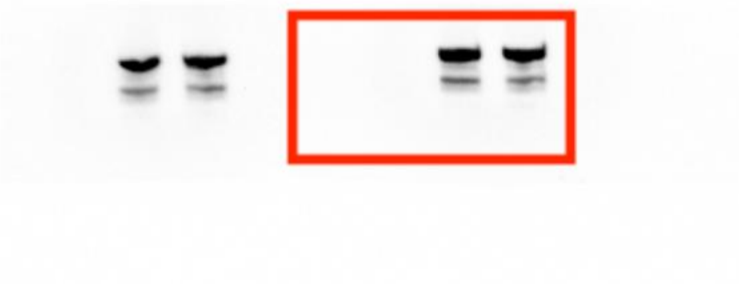

immunoblot of MHC1 and GAPDH

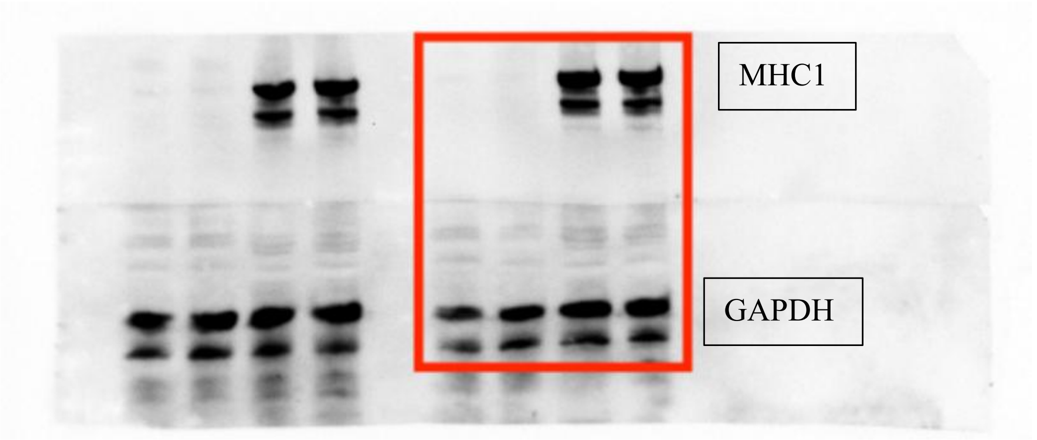

Supplement: Supplementary file 3 — Original western blots [file 41420_2023_1365_MOESM3_ESM.pdf]
